# Supplementation with a cranberry extract favors the establishment of butyrogenic guilds in the human fermentation SHIME system

**DOI:** 10.20517/mrr.2024.17

**Published:** 2024-06-14

**Authors:** Valentina Cattero, Charlène Roussel, Jacob Lessard-Lord, Denis Roy, Yves Desjardins

**Affiliations:** ^1^Institute of Nutrition and Functional Foods (INAF), Faculty of Agriculture and Food Sciences, Laval University, Quebec City G1V 0A6, Quebec, Canada.; ^2^Centre Nutrition, Santé et Société (NUTRISS), INAF Laval University, Quebec City G1V 0A6, Quebec, Canada.; ^3^Excellence Research Chair on the Microbiome-Endocannabinoidome Axis in Metabolic Health, Laval University, Quebec City G1V 0A6, Quebec, Canada.

**Keywords:** Proanthocyanidins, gut microbiota, bifidogenic effect, mucus, *Akkermansia muciniphila*, butyrate, M-SHIME fermentation system

## Abstract

**Background:** Proanthocyanidins (PAC) and oligosaccharides from cranberry exhibit multiple bioactive health properties and persist intact in the colon post-ingestion. They display a complex bidirectional interaction with the microbiome, which varies based on both time and specific regions of the gut; the nature of this interaction remains inadequately understood. Therefore, we aimed to investigate the impact of cranberry extract on gut microbiota ecology and function.

**Methods:** We studied the effect of a cranberry extract on six healthy participants over a two-week supplementation period using the *ex vivo* artificial fermentation system TWIN-M-SHIME to replicate luminal and mucosal niches of the ascending and transverse colon.

**Results:** Our findings revealed a significant influence of cranberry extract supplementation on the gut microbiota ecology under *ex vivo* conditions, leading to a considerable change in bacterial metabolism. Specifically, *Bifidobacterium adolescentis* (*B. adolescentis*) flourished in the mucus of the ascending colon, accompanied by a reduced adhesion of *Proteobacteria*. The overall bacterial metabolism shifted from acetate to propionate and, notably, butyrate production following PAC supplementation. Although there were variations in microbiota modulation among the six donors, the butyrogenic effect induced by the supplementation remained consistent across all individuals. This metabolic shift was associated with a rise in the relative abundance of several short-chain fatty acid (SCFA)-producing bacterial genera and the formation of a consortium of key butyrogenic bacteria in the mucus of the transverse colon.

**Conclusions:** These observations suggest that cranberry extract supplementation has the potential to modulate the gut microbiota in a manner that may promote overall gut health.

## INTRODUCTION

The American cranberry (*Vaccinium macrocarpon*) is a fruit containing various bioactive components, particularly known for being a major source of proanthocyanidins (PAC), which are oligomers and polymers of flavan-3-ols. Many *in vitro*, preclinical, and clinical studies have shown that PAC offers health advantages beyond merely guarding against urinary tract infections. Studies have demonstrated anti-inflammatory, cardioprotective, neuroprotective, immunomodulatory, lipid-lowering and anti-obesity, antidiabetic, and anticancer properties^[1-6]^. However, the exact mechanisms by which PAC delivers these health benefits are still not fully understood^[2]^. Increasing evidence suggests a link between the health benefits of PAC and the gut microbiota. Indeed, due to their high degree of polymerization and complex structures, 95% of PAC are not absorbed in the small intestine, are poorly metabolized in the small intestine and reach the colon intact, where they can interact with the microbiota^[7-9]^.

Furthermore, recent research has revealed that cranberries are rich in potentially bioactive oligosaccharides. In fact, it has been suggested that (poly)phenol-rich extracts on the market could actually contain approximately 20% (*w/w*) oligosaccharides, primarily arabinoxyloglucans and pectic oligosaccharides^[10]^. These complex carbohydrates can withstand gastric digestion and bind to (poly)phenols, favoring their transport to the colon for subsequent utilization by the gut microbiota^[10,11]^.

Recent studies have emphasized the capacity of (poly)phenols to impact the composition and functionality of the gut microbiota^[8,12,13]^. Our group has recently proposed that (poly)phenols exhibit a “duplibiotic” action. This refers to their capacity to alter the composition of the intestinal microbiota by exhibiting antimicrobial properties against specific pathobionts, while also having a prebiotic effect through their metabolism by the microbiota^[14]^. However, this bidirectional interaction has not been fully described, and the way in which PAC/oligosaccharides influence the gut microbiota remains inadequately characterized. We are just at the initial stages of identifying the commensal bacterial species responsible for PAC catabolism and understanding their role in (poly)phenol metabolism^[8]^.

Recent studies have begun to explore the ways in which PAC might influence the composition of the gut microbiota and its potential effects on the health of the host. This includes *in vitro* and *in vivo* studies, as well as some clinical trials involving PAC supplementations from different sources (e.g., grape^[15]^, aronia berry^[16]^, blueberry^[17]^, avocado^[18]^). These studies have consistently observed a stimulation of bifidobacteria and lactobacilli^[19-22]^. Moreover, *Roseburia* species and *Faecalibacterium praustnitzii* seem to flourish after PAC supplementation, while the response of *Enterobacteriaceae* is not consistent across studies^[19,22]^. PAC also favorably affect the bacteria *Akkermansia muciniphila*^[19-22]^. This bacterium is considered a next-generation probiotic and prefers the intestinal mucosal environment, where it can positively impact immunoprotection through stimulation of mucus turnover and enhancement of gut barrier function^[17,23-25]^. There is increasing interest in the microbiota associated with the intestinal mucus layer, as this ecological niche is essential for preventing direct contact between harmful bacterial species and epithelial cells, thus playing a critical role in maintaining gut homeostasis^[20,26]^.

We surmise that a cranberry extract, rich in both PAC and oligosaccharides, beneficially alters the gut microbiota and fosters the development of a niche conducive to beneficial gut microbiota. The objective of this study was to investigate the impact of cranberry extract on the gut microbiota of six healthy individuals, utilizing the artificial fermentation system TWIN-M-SHIME. This is an established *in vitro* system able to simulate the microbial ecosystem of the human gut dynamically operating two systems in parallel at the same time, remaining faithful to the original donor’s microbiota^[27]^. The system also included a simulated intestinal interface providing mucus-associated microbiotas mimicking mucosal microbial colonization. Specifically, the study sought to examine how cranberry extract influences the composition of commensal bacteria *ex vivo*, both within the lumen and in the mucus layer of the ascending and transverse section of the colon. In addition, we analyzed the production of fermentation by-products, specifically short-chain fatty acids (SCFA), to understand potential shifts in bacterial metabolic functions. Finally, we delved deeper into the ecological interactions through the examination of co-occurrence networks.

Our study revealed that cranberry extract supplementation significantly impacted the gut microbiota ecology, leading to a substantial shift in bacterial metabolism. Notably, *Bifidobacterium adolescentis* (*B. adolescentis*) thrived in the mucus of the ascending colon. The overall bacterial metabolism shifted from acetate to propionate and, especially, butyrate production. This shift was associated with the establishment of a consortium of keystone butyrogenic bacteria in the mucus of the transverse colon.

## METHODS

### Supplement

A cranberry extract (Prebiocran^TM^) was supplied by Symrise (Diana Food Canada Inc.). The composition of this (poly)phenolic extract rich in PAC can be found in previously published studies^[28,29]^. The dose was set to 86.8 mg PAC/day/donor, which corresponds to two capsules per day, or the equivalent of consuming 60 g of fresh cranberry. The supplement delivered 125 mg of oligosaccharides and 109.3 mg of total polyphenols per day. The oligosaccharide composition mainly consisted of glucose (58%), arabinose (24%), xylose (10%), and galactose (4%). Polyphenols were mainly flavan-3-ols (75%), followed by flavonols (13%), phenolic acids (7%), and anthocyanins (5%). Flavan-3-ols monomers consisted of 0.1293 mg of catechin, 0.1865 mg of epicatechin, and 4.46 mg of procyanidins A2 per day. Procyanidins B2 monomers were not detected. Flavan-3-ols in cranberry extract had a mean degree of polymerization of 5 and were composed of 33% procyanidin A2 units. Prebiocran^TM^ is standardized by Symrise at min. 30% (poly)phenols (Folin-Ciocalteu method - gallic acid equivalent).

### TWIN-M-SHIME^®^ system

**Figure 1:**
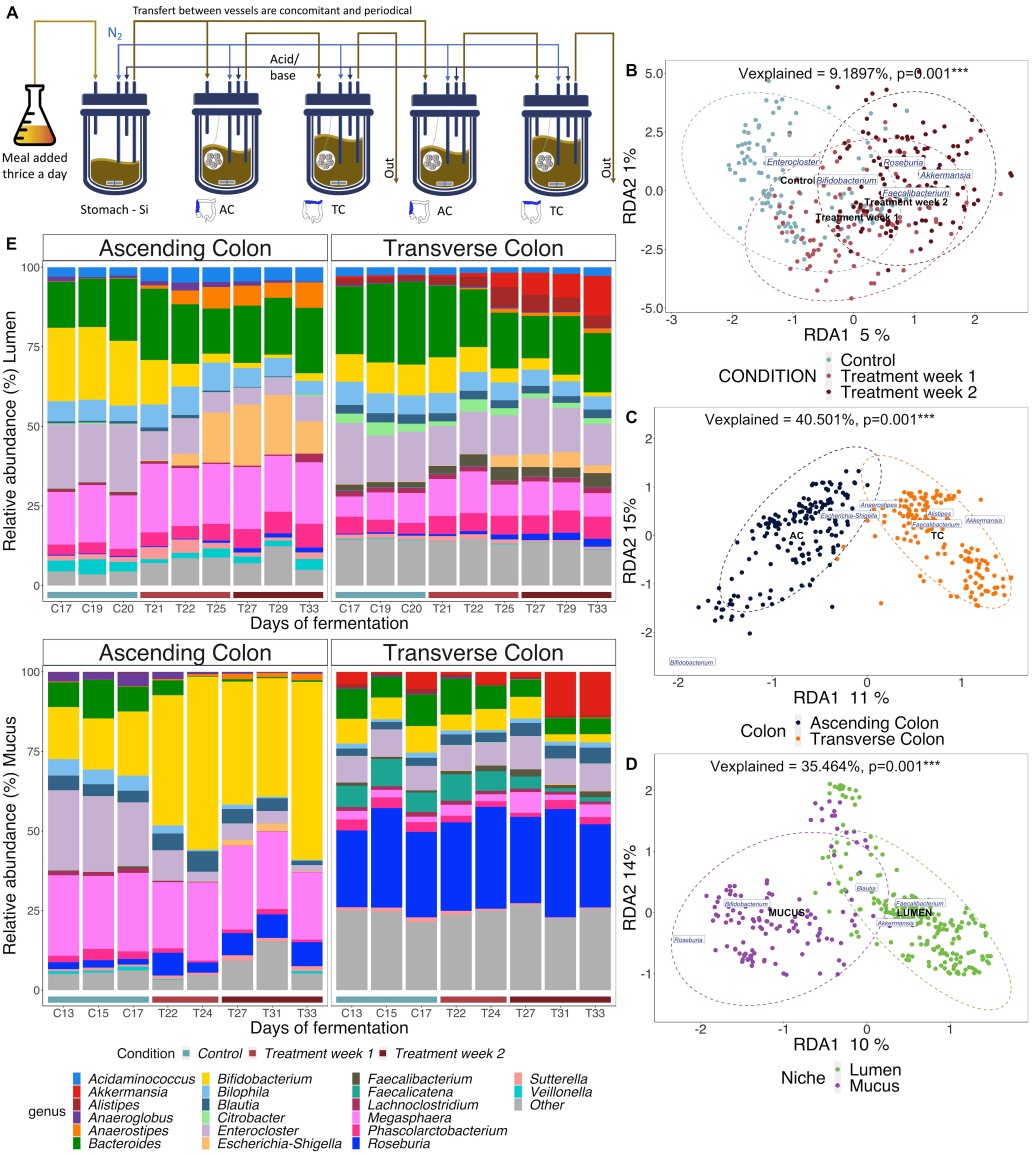
Cranberry extract, colon regions and lumen/mucus niches dynamically affect the microbiota in the TWIN-M-SHIME. (A) Schematic representation of the TWIN-M-SHIME® system setup. Each colonic region was inoculated in duplicate for each donor; (B-D) Partial distance-based redundancy analysis (db-RDA) of the microbial community composition based on 16S rRNA gene amplicon sequencing showing that treatment (B), colon region (C) and lumen/mucus niche (D) explain the differences in the microbiota. Explanatory variables are indicated in black (as shown in legend) and response variables are labeled in blue (genera abundances, only the principal explanatory variables are displayed for visibility). Percent on x- and y-axes indicate contribution to the total variance, while “*Vexplained*” corresponds to the variability of the gut microbiota composition explained by the variables (^***^P < 0.001 significance) assessed from the distance matrix PERMANOVA; (E) Stacked bar plots of gut microbiota genus-level relative abundance during specified days of fermentation: following two weeks of stabilization and one week of control period, the extract was added from the 21st day, at 86.8 mg PAC/day/donor. The graphic indicates the one-week control period (C; blue line), the first week of cranberry treatment (T; light red line), and the second week of cranberry treatment (T; dark red line). The top graph shows the proportion of the 20 most abundant genera in the lumen, while the bottom one of the mucus in the respective ascending and transverse colon. The stacked bar plots show the mean composition of six donors in duplicate. PAC: Proanthocyanidins.

The TWIN-M-SHIME® simulates the microbial ecosystems of the human large intestine^[27]^. The first vessel replicated the stomach and the small intestine according to a fill-and-draw principle with the delivery of nutritional medium and pancreatic/bile juices, followed by vessels representing two repetitions of the ascending and transverse colon [Figure 1A] with fixed volumes. The detailed functioning of the system has been published^[29]^ and it is described in Supplementary Data.

A total of six healthy individuals with no recent history of antibiotic usage provided fecal samples across three experiments. Consent for fecal donation was obtained under registration number 2019-312 (Laval University, Canada). The selection of these donors was based on a prior screening of cranberry PAC urinary metabolite production on 11 subjects. Information regarding the gender, ethnicity, and dietary habits of the six donors is included in Supplementary Table 1. The candidates were selected as the most contrasting producers and non-producers of PAC urinary metabolites (phenyl-γ-valerolactones). The methods for fecal inoculum collection, preparation, and inoculation, along with details on the system’s overall operation, replacement of the mucin carrier, and the composition of the media, were outlined in a prior study^[29]^ and are detailed in Supplementary Data.

The overall TWIN-M-SHIME® fermentation process lasted 33 days, consisting of a 12-day period to allow the microbiota to stabilize and adapt to the *in vitro* conditions, and a 7-day control phase in which the microbiota are only fed the SHIME feed and the pancreatic juices. The samplings during this period (3) are considered the control to assess the effect of the cranberry supplement fed to the system during the following 14 days. The treatment involved the supplementation of a cranberry extract (Prebiocran^TM^), providing 86.6 mg of flavan-3-ols (determined by phloroglucinolysis method^[30,31]^) per day, administered in the SHIME stomach with the second meal of the day.

### DNA extraction

DNA extraction of the SHIME samples was previously described^[29,32-34]^. In the case of mucus microcosm samples, an additional step using sputolysin 0.1 M was included to enhance DNA yield and disrupt the disulfide bonds present in the mucins^[35]^. Finally, a purification step of DNA was added using the OneStep^TM^ PCR Inhibitor Removal Kit (Zymo Research Corp., Orange, CA, United States) following the manufacturer’s protocol.

### SCFA production

The effluents from the colon vessels of the SHIME were subjected to centrifugation at 18,000 × *g* for 8 min at 4 °C. SCFA were extracted using diethyl ether as previously described^[29]^ and subsequently analyzed using a gas chromatograph system connected to a flame ionization detector. The concentration of SCFA was quantified in mM units.

### Microbial community analysis

Following the DNA extraction from the SHIME samples, we performed next-generation sequencing on the V3-V4 region (341F-805R) of the 16S rRNA gene. For bioinformatics analysis, the Divisive Amplicon Denoising Algorithm (DADA2) workflow from the dada2 R package 1.26 was utilized^[29]^. When species identification using the SILVA database was incomplete, a manual refinement was conducted using the RDP SeqMatch classifier and NDPI Microbes BLASTN tools.

### Statistical analysis

Statistical analysis was performed using R (v. 4.3.0). The Chao and Shannon indices were computed to assess the evolution of microbial community α-diversity between conditions using vegan package (v. 2.6-4) and ggpubr (v. 0.6.0) to compare means statistics using the Kruskal-Wallis test. The db-RDA method was used to determine the influence of treatment over time, gut regions, donors, and niches. The significance of group separation was assessed using PERMANOVA. DESeq2 package (v. 3.17) was used to find statistically significant differences in species/genera abundance between control and supplementation periods. *P*-values were adjusted for multiple testing using the Benjamini-Hochberg post-hoc test. Significant differences were visualized using box plots and volcano plots, as previously described^[36]^. The co-occurrence matrices were constructed with the SPIEC-EASI R package (v. 1.1.0)^[37]^ based on the filtered and normalized abundance matrix of each condition, colon region, and niche. The resulting adjacency matrices were then uploaded in Cytoscape (v. 3.10.0) to visualize the microbial networks and analyze the networks, allowing for the keystone identification based on the high degree, low betweenness centrality, and high closeness centrality parameters, as previously described^[38]^. To evaluate the impact of the cranberry extract on the metabolic activity (SCFA), statistical analysis was conducted using the Kruskal-Wallis rank sum test with post hoc Dunn’s test. The Spearman’s correlations between the concentration of the three major SCFA and the relative abundance of the main bacterial genera were calculated using the corrr R package (v. 0.4.4).

## RESULTS

### The response of gut microbiota to the cranberry extract varies based on the specific colon region and ecology

The TWIN-M-SHIME® model was used to study changes in the microbial community composition in the ascending and transverse colon regions, encompassing both luminal and mucus content, over a two-week period of supplementation with cranberry extract, compared to a one-week control period [Figure 1A].

Six different healthy donors were tested over three different experiments, and their microbiota was first analyzed separately (data available in Supplementary Figures 1-5) and then pooled to evaluate the effect on the overall population. Distance-based redundancy analysis (db-DRA) confirmed that the supplementation had a strong impact on the overall differences observed in the microbial composition at the genus level (representing 5.5% of the total variation, *P* = 0.001, PERMANOVA) [Figure 1B]. The gut modulation effect was gradual over time, and therefore, the two weeks of supplementation were treated separately in subsequent analysis [Figure 1B]. As expected, the inter-individual variability accounted for a substantial percentage of the dissimilarities (35.9%, *P* = 0.001), but there was no apparent clustering between the donors [Supplementary Figure 6]. The colon regions (12.1%, *P* = 0.001) and the lumen/mucus environments (10.2%, *P* = 0.001) were significant explanatory variables as well [Figure 1C and D]; thus, we evaluated the average shifts in the relative abundance of the microbial community under supplementation separately for each specific region/environment [Figure 1E]. Distinct microbiota signatures were identified based on the top 20 most abundant genera. The genera *Megasphaera* and *Bifidobacterium* preferred the ascending colon region (Figure 1E, top left), while the transverse colon displayed a more diverse microbiota, including genera like *Akkermansia*, as anticipated (Figure 1E, top right). The *Roseburia* and *Bifidobacterium* genera effectively colonized a significant portion of the mucus environment in the transverse and ascending colon, respectively (Figure 1E, bottom).

### The cranberry extract induces a stronger microbiota modulation in the ascending colon than in the transverse colon

Supplementation with cranberry extract led to a notable enhancement in α-diversity species richness, as measured by the Chao index, exclusively in the lumen (*P* = 0.05, Kruskal-Wallis) and mucus (*P* = 0.001) environments of the ascending colon. However, there was a simultaneous decline in species evenness in the mucus of the same colon region (*P* = 0.001), according to the Shannon index, as detailed in Table 1.

**Table 1:** Changes in species richness and evenness induced by the supplementation with the cranberry extract

**Condition**	**Mean Chao index (X10000)**	**Mean Shannon index**
Lumen ascending colon control week	27.07 (± 20.28)	2.95 (± 0.54)
Lumen ascending colon treatment week 1	30.86 (± 1.84)	3.03 (± 0.66)
Lumen ascending colon treatment week 2	32.45 (± 20.96)^*^	2.79 (± 0.56)
Mucus ascending colon control week	33.25 (± 19.36)	3.13 (± 0.82)
Mucus ascending colon treatment week 1	54.27 (± 25.90)^*^	2.60 (± 1.33)^**^
Mucus ascending colon treatment week 2	61.00 (± 24.06)^***^	2.16 (± 0.42)^***^
Lumen transverse colon control week	51.23(± 8.60)	4.12 (± 0.18)
Lumen transverse colon treatment week 1	51.89 (± 6.46)	4.11 (± 0.36)
Lumen transverse colon treatment week 2	50.48 (± 13.65)	3.99 (± 0.38)
Mucus transverse colon control week	42.34 (± 21.90)	3.68 (± 0.67)
Mucus transverse colon treatment week 1	38.98 (± 22.83)	3.80 (± 0.48)
Mucus transverse colon treatment week 2	41.55 (± 24.24)	3.70 (± 1.02)

Changes in the Shannon diversity indices were examined as the gut regions/environments transitioned from a 1-week control phase to two subsequent 1-week cranberry extract supplementation phase, with six donors. ^*^P = 0.05; ^**^P = 0.01; ^***^P = 0.001.

**Figure 2:**
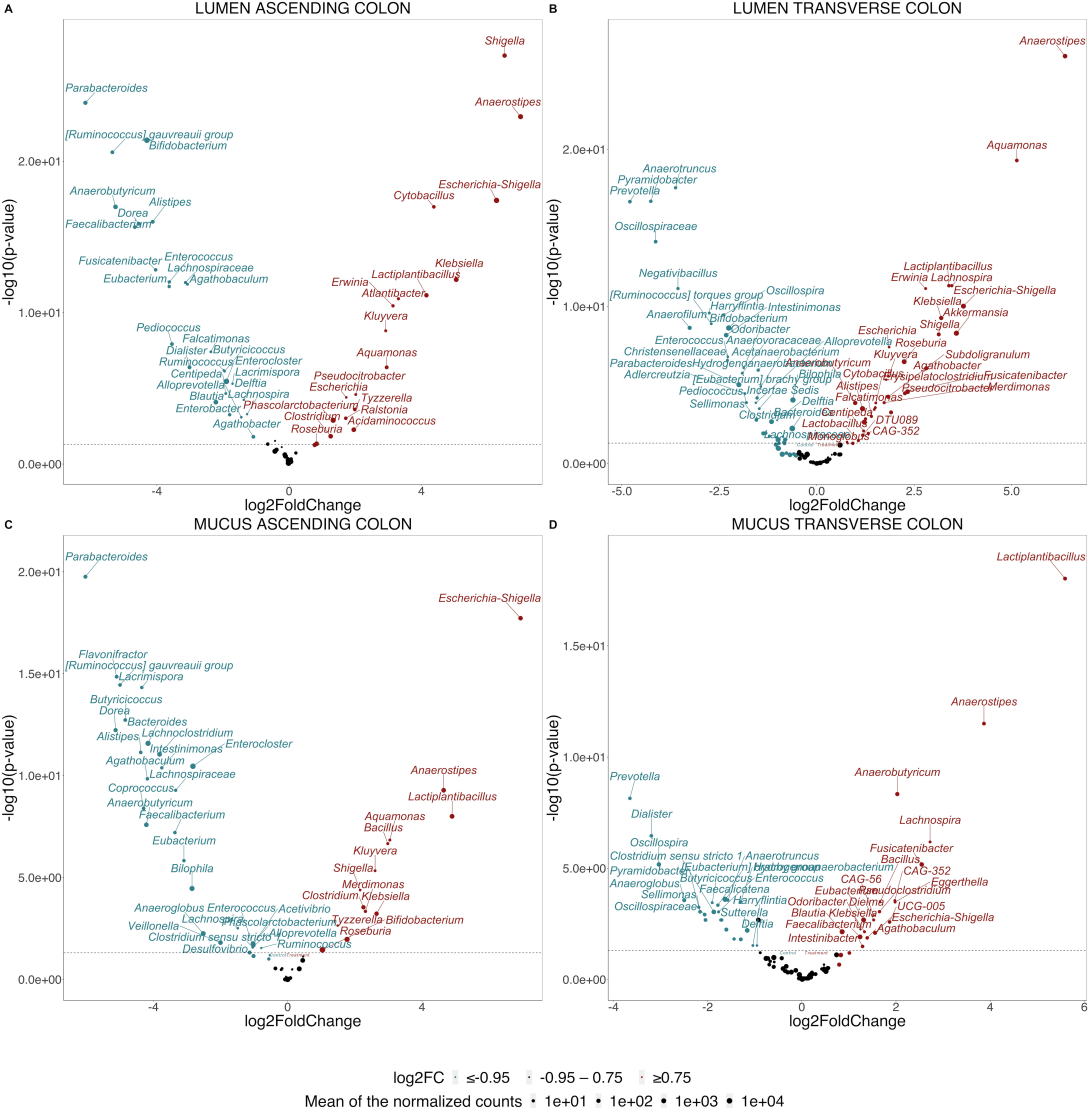
The supplementation with a cranberry extract exhibits distinct effects on microbiotas across different colon regions. Volcano plots displaying the significantly enriched genera after supplementation as determined using a Wald test with Benjamini-Hochberg multiple testing correction in the lumen (A) and mucus (C) of the ascending colon and the lumen (B) and mucus (D) of the transverse colon, respectively. Deseq2 analysis of log2 fold-changes displays on the left of the x-axis (in blue) the genera depleted after the two weeks of supplementation, while on the right (in red), the ones enriched after the two weeks of supplementation. The y-axis shows the log-transformed adjusted *P*-value with the dashed line indicating the α = 0.05 significance threshold. Statistical differences between the control and the second week of supplementation were determined using a Wald Test with Benjamini-Hochberg multiple correction test.

The DESeq analysis additionally revealed significant variations in the relative abundance of numerous genera within the luminal and mucus microbiota of the ascending colon when comparing the control week to the second week supplemented with the cranberry extract (Figure 2A-C, Supplementary figures 7 and 8 for donor-specific results).

Strong enrichment (> 4 log2FC, *P* < 0.05, Wald test) of genera belonging to *Proteobacteria (Shigella*, *Escherichia-Shigella*, and *Klebsiella*) (6/6 donors), *Anaerostipes* (2/6 donors), and *Lactiplantibacillus* (4/6 donors) was specifically observed in the luminal ascending colon and, to a lesser but still significant extent, *Phascolarctobacterium* (3/6 donors)*, Acidaminococcus* (1/6 donors)*,* and *Roseburia* (1/6 donors) were also stimulated, while *Bifidobacterium* (5/6 donors), *Parabacteroides* (3/6 donors), and *Faecalibacterium* (1/6 donors) were depleted (> -4 log2FC, *P* < 0.05) [Figure 2A]. In the mucus environment of the same region, the genera *Escherichia-Shigella* (3/4 donors), *Anaerostipes* (3/4 donors), and *Lactobacillus* (2/4 donors) responded positively to the treatment (> 4 log2FC, *P* < 0.05), alongside *Bifidobacterium* (4/4 donors) that strongly colonized the mucus [Figure 2C]. Although no change in α-diversity was observed in the transverse colon following supplementation, DESeq analysis revealed that several genera were affected in both the luminal and mucus environments of this region (Figure 2B-D, Supplementary figures 7 and 8 for donor-specific results). In the lumen, the relative proportions of *Anaerostipes* (3/6 donors), *Akkermansia* (3/6 donors), and *Lactiplantibacillus* (4/6 donors) increased after supplementation alongside some *Proteobacteria* genera (*Aquamonas*, *Shigella*, *Escherichia-Shigella*, and *Klebsiella*) (> 3 log2FC, *P* < 0.05) (5/6 donors), while *Prevotella* (3/6 donors) and some genera of the *Oscillospiraceae* family (*Oscillospiraceae_unknown*, *Anaerotruncus*, and *Anaerofilum*) (6/6 donors) were depleted (> -3 log2FC, *P* < 0.05) [Figure 2B]. In the mucus niche, *Lactiplantibacillus* (2/4 donors) and *Anaerostipes* (2/4 donors) were favored by the supplementation (> 3 log2FC, *P* < 0.05), whereas *Prevotella*, *Dialister*, and *Oscillospira* (4/4 donors) were blunted (> -3 log2FC, *P* < 0.05) [Figure 2D].

### *Bifidobacterium* genus responds differently to the cranberry extract in the ascending colon, showing a preference for *B. adolescentis* in the mucus environment

**Figure 3:**
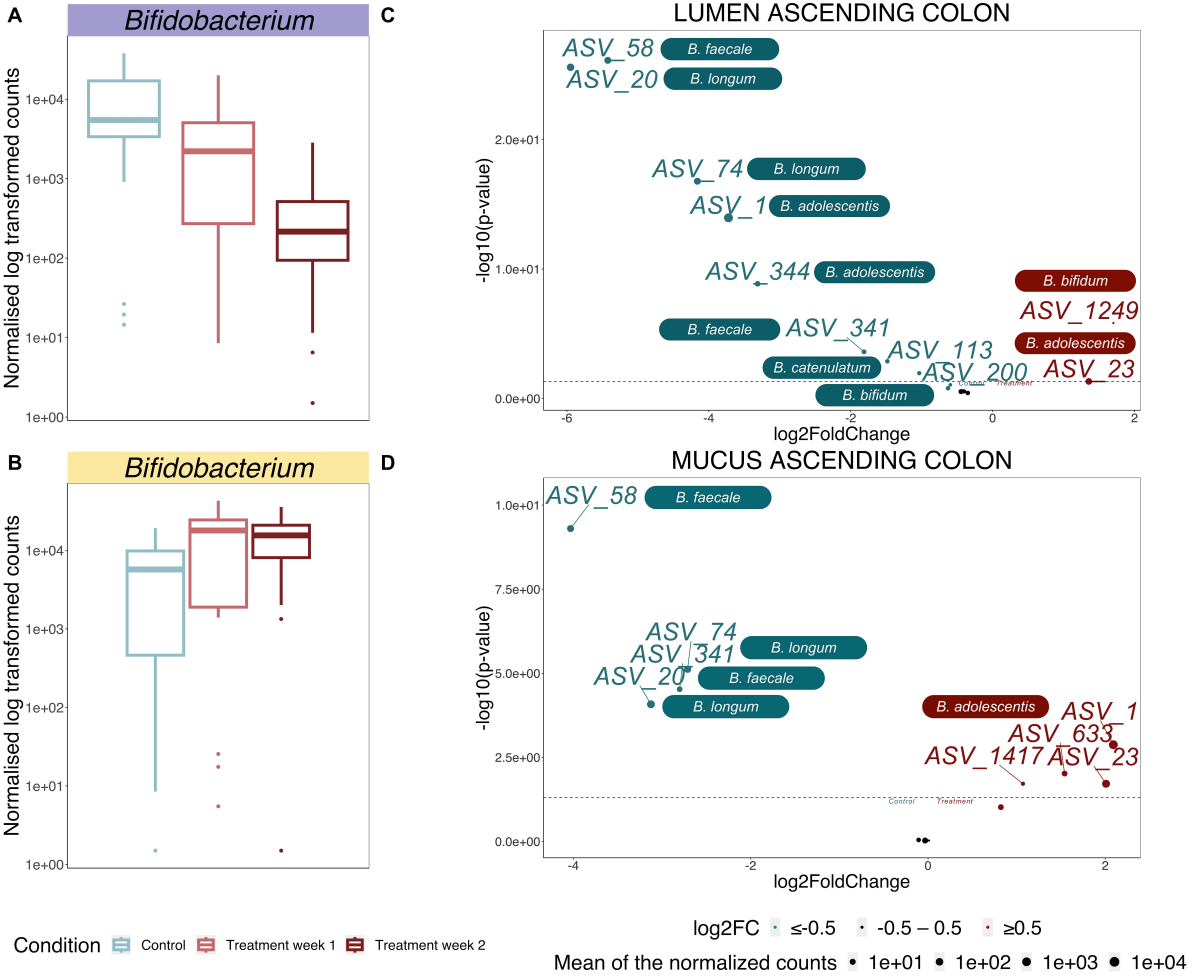
*Bifidobacterium* species shifts induced by the supplementation with a cranberry extract in the ascending colon. (A and B) *Bifidobacterium* genus behavior following the supplementation in the lumen (A) and mucus (B), respectively, as evaluated by Deseq2 analysis. The changes between the control and the second week of supplementation are significantly different at α = 0.05, as determined using a Wald Test with Benjamini-Hochberg multiple testing correction; (C and D) Volcano plots showing the ASVs assigned to *Bifidobacterium* species significantly affected by the supplementation as determined by the Wald test with Benjamini-Hochberg multiple testing correction in the lumen (C) and mucus (D) of the ascending colon. Deseq2 analysis of log2 fold-changes displays on the left of the x-axis (in blue) the ASVs depleted after the two weeks of supplementation, while on the right (in red), the ones enriched after the two weeks of supplementation. Each ASV sequence was assigned to the corresponding species based on RDP classifier. The y-axis shows the log-transformed adjusted *P*-value with the dashed line indicating the α = 0.05 significance threshold.

The cranberry extract had a significant impact on the *Bifidobacterium* genus in the ascending colon, showing a distinctive and varied response depending on the intestinal niche observed [Figure 3A and B].

In the lumen, the relative abundance of this species gradually decreased over the two-week supplementation period (5/6 donors) (*P* < 0.05) [Figure 3A], while it exhibited a remarkable propensity to flourish within the mucus of the same colon region (4/4 donors) (*P* < 0.05) [Figure 3B], attaining roughly 40% of the total relative abundance during the second week of treatment (Figure 1E, bottom left). Upon closer examination, the RDP and NBLAST assignations revealed that several ASVs (ASV_1, ASV_23, ASV_633, ASV_1417) associated with the *B. adolescentis* species were the main colonizers, making up over 50% of the *Bifidobacterium* species found in the mucus [Supplementary Figure 9]. Several other ASVs associated with different *Bifidobacterium* species [*B. faecale* (ASV_58, ASV_341), *B. longum* (ASV_20, ASV_74), *B. catenulatum* (ASV_113), *B. bifidum* (ASV_200)] were depleted in response to the supplementation in both environments of the same colon region [Figure 3C and D]. Only one other ASV associated with the *B. bifidum* species (ASV_1249) was enriched after the two-week supplementation in the lumen (*P* < 0.05) [Figure 3C].

### The extent to which the cranberry extract stimulates known PAC-degrading bacterial species is largely influenced by the donors’ microbiota baseline composition

So far, only a limited number of gut bacterial strains have been shown to metabolize PAC, namely *Adlercreutzia equolifaciens*, *Eggerthella lenta*, *Slackia equolifaciens*, *Flavonifractor plautii*, *Eubacterium oxidoreducens*, and *Lactiplantibacillus plantarum* [Table 2].

**Table 2:** Characterization of species known to be implicated in the PAC metabolism

**Species**	**Phylum (class)**	**Reaction**	**Ref.**
*Adlercreutzia equolifaciens*	*Actinobacteria* (*Coriobacteriia*)	Dehydroxylation C4’ C-ring cleavage	[8,39]
*Eggerthella lenta* [ASV_1702, (100%)]	*Actinobacteria* (*Coriobacteriia*)	C-ring cleavage	[8,39-41]
*Slackia equolifaciens*	*Actinobacteria* (*Coriobacteriia*)	C-ring cleavage	[8,39]
*Flavonifractor plautii* [ASV_655, (98.02%)]	*Firmicutes* (*Clostridia*)	Further degradation	[8,41,42]
*Eubacterium oxidoreducens*	*Firmicutes* (*Clostridia*)	A-ring cleavage	[8,43]
*Lactiplantibacillus plantarum* [ASV_69 (100%), ASV_238 (99.77%), ASV_617 (91.86%), ASV_1511 (99.65%), ASV_1979 (91.63%)]	*Firmicutes* (*Bacilli*)	C-ring cleavage	[8,44]

Principal bacterial species identified in the gut that are capable of metabolizing flavan-3ols are listed along with the corresponding ASVs and NCBI BLASTN percent identity score. PAC: Proanthocyanidins.

**Figure 4:**
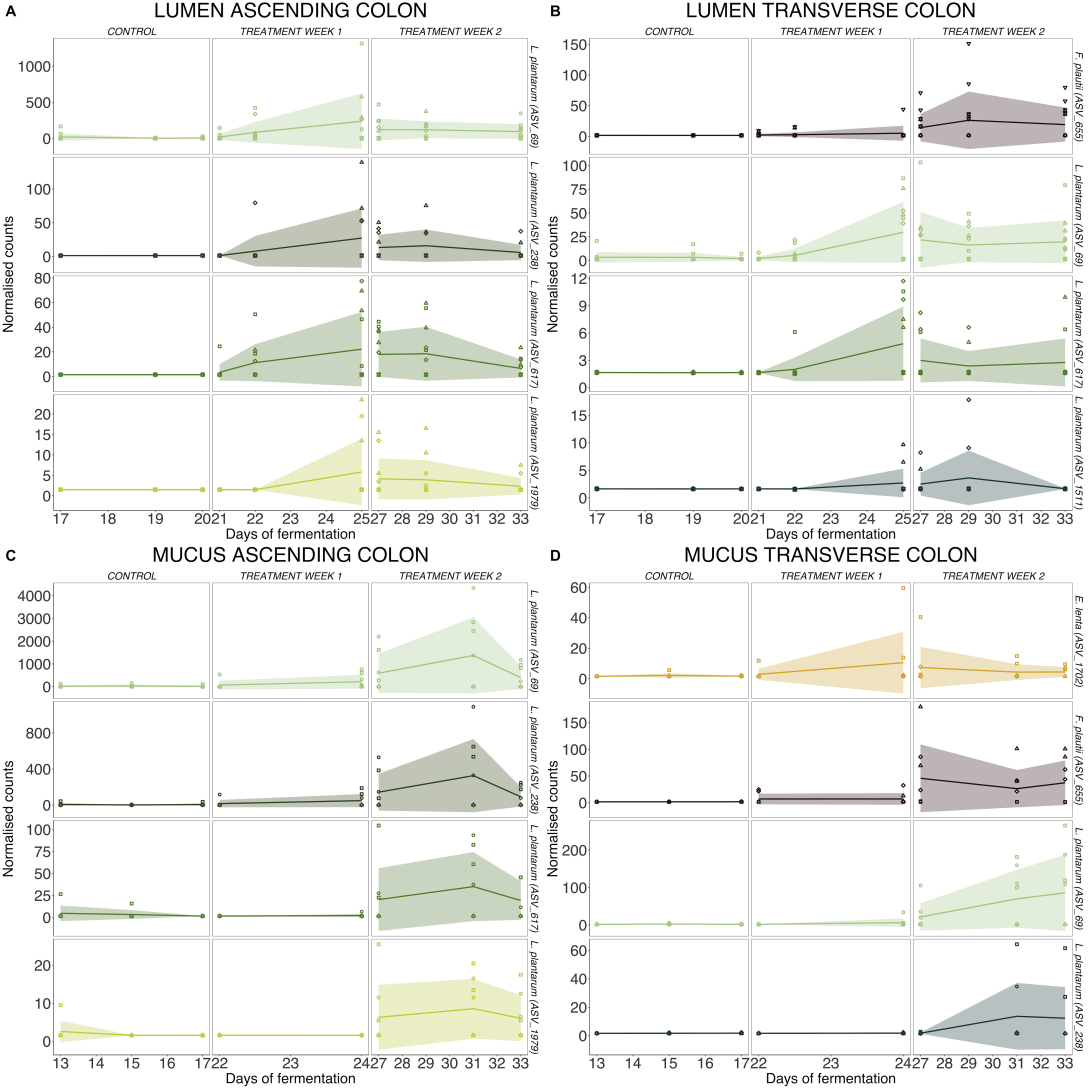
Follow-up of bacterial species known to be involved in the PAC metabolism. Evolution of bacteria species potentially involved in PAC metabolism during the TWIN-M-SHIME® fermentation for the six donors. Deseq2 analysis was performed separately in the lumen of the (A) ascending and (B) transverse colon and in the mucus of the (C) ascending and (D) transverse colon. Statistical differences between the control and cranberry extract supplementation across gut regions were determined using a Wald Test with Benjamini-Hochberg multiple testing correction. Only species with statistical differences (α = 0.05) between the control and the second week of treatment are presented. PAC: Proanthocyanidins.

While 16S rRNA gene sequencing limits our ability to identify organisms beyond the species level, we have closely monitored the progression of ASVs assigned to these species throughout the fermentation process. This approach has enabled us to semiquantitatively track their presence and response to the supplementation. The DESeq analysis revealed that both the presence of these species and their response to the supplementation are significantly influenced by the individual donor and the specific region or niche within the intestinal environment [Figure 4].

In the case of the ascending colon, various ASVs linked to the species *L. plantarum*/*L. fabifermentans* (such as ASV_69, ASV_238, ASV_617, ASV_1979) showed a significant positive response to the cranberry extract supplementation. This response was observed both in the lumen and in the mucus environment [Figure 4A-C]. In the transverse colon, we also observed a significant growth of *L. plantarum/fabifermentas* (ASV_69, ASV_238, ASV_617, ASV_1511), alongside *F. plautii* (ASV_655) and *E. lenta* (ASV_1702) that bloomed in the mucus of only one specific donor [Figure 4B and D].

### The administration of cranberry extract led to the development of butyrogenic keystone bacterial guilds in both the luminal and mucus regions of the transverse colon, resulting in a marked increase in butyrate production

**Figure 5:**
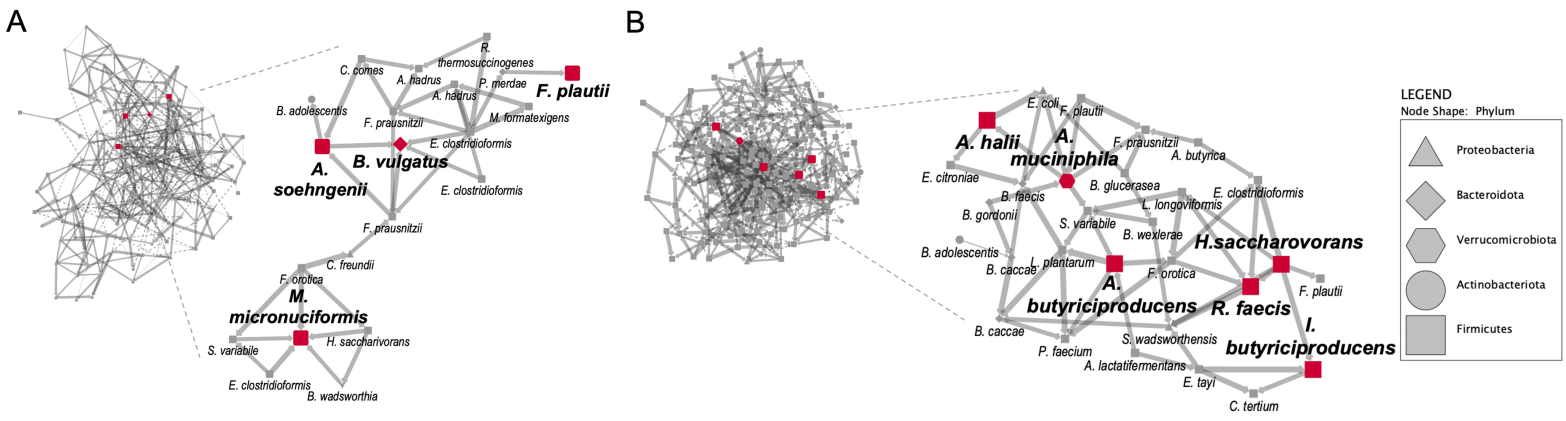
Establishment of butyrogenic guilds and identification of keystone species following the cranberry extract supplementation. (A and B) Networks of significant co-occurrences between the bacterial species (ASV) based on 16S rRNA gene amplicon sequencing in the transverse colon for the lumen (A) and the mucus (B) during the second week of supplementation. The nodes are shaped according to the corresponding phylum and the edges indicate a positive (full lines) or negative (dotted lines) correlation between the network nodes. From the networks, bacterial guilds are displayed. Red nodes indicate the ASVs identified as keystone species according to network analysis of high *degree*, low *betweenness centrality*, and high *closeness centrality* simultaneously significant at the α = 0.05 interval.

To determine strong bacterial associations, we evaluated the evolution of co-occurrence interactions over the fermentation period. By organizing the ASV table into subsections for the two colon regions, the two environments, and the week of control/treatment, we conducted an analysis that resulted in 12 distinct networks [Supplementary Figure 10]. The topological properties of these co-occurrence networks are available in Supplementary Table 2. This method represents ASVs as interconnected nodes when a co-occurrence relationship is statistically significant between them. The visualization of the resulting networks showed that the cranberry extract improved the co-occurrence patterns after two weeks of supplementation, resulting in more frequent significant correlations between different ASVs [Supplementary Figure 10]. By analyzing these co-occurrence networks, we were able to identify two bacterial guilds specific to the lumen and the mucus environments of the transverse colon [Figure 5], despite not being able to reveal interesting patterns in the ascending colon [Supplementary Figure 10A-F].

Each guild revolved around key members (i.e., keystone species), identified through three topological network parameters: high *degree*, low *betweenness centrality*, and high *closeness centrality* [Supplementary Table 3], as previously shown^[38]^. *Megasphaera micronuciformis* (ASV_6), *Bacteroides vulgatus* (ASV_25), *F. plautii* (ASV_40), and *Anaerobutyricum soehngenii* (ASV_68) were identified as keystone species in the lumen of the transverse colon during the last week of supplementation [Figure 5A]. In the mucus region of the transverse colon, several well-known butyrate-producing species, namely *A. muciniphila* (ASV_18), *R. faecis* (ASV_111), *Intestinimonas butyriciproducen*s (ASV_205), *Anaerobutyricum hallii* (ASV_231), *Agathobaculum butyriciproducen*s (ASV_1052), and *Hydrogenoanaerobacterium saccharovorans* (ASV_1096), were identified as keystone species [Figure 5B]. These species are recognized butyrate producers, which is consistent with SCFA results.

**Figure 6:**
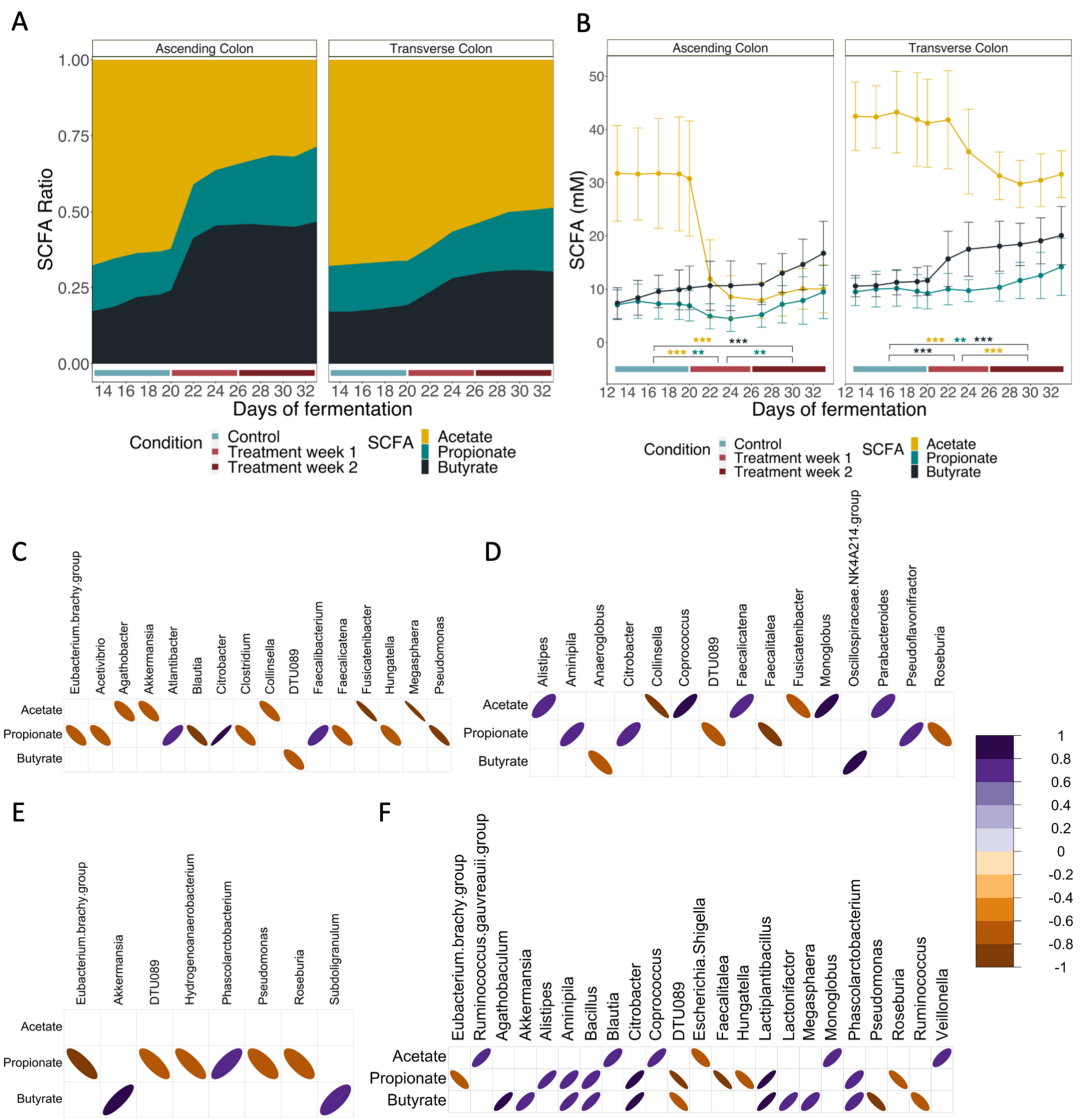
SCFA modulation by cranberry extract supplementation and correlations with bacterial genera. (A) Mean ratio and (B) mean concentration ±SD over time of the three major SCFA for the six donors in the ascending and transverse colon during the control week (blue line) and the two subsequent weeks of treatment (week 1: light red, week 2: dark red). Statistically significant differences between control and treatment period are denoted for *P* < 0.05 (^*^), *P* < 0.01 (^**^), and *P* < 0.001 (^***^) as determined by Kruskal-Wallis followed by post-hoc Dunn’s test. (C-F) Spearman correlations between the three major SCFA and bacterial genera in the transverse colon. Correlations were performed in the lumen (C and E) and mucus (D and F) environments separately during the first (C and D) and second week (E and F) of supplementation. Only negative and positive significant correlations (*P* < 0.05 Benjamini-Hochberg adjusted) that are greater than a -0.7 or 0.7 coefficient are shown, denoted in shades of orange and purple, respectively. SCFA: Short-chain fatty acid.

In fact, in both colon regions, butyrate concentrations increased progressively during fermentation and were significantly higher after two weeks of supplementation (6/6 donors) (*P* < 0.001), even surpassing acetate concentrations and ratios in the ascending colon [Figure 6A and B].

During the control period, both regions of the colon maintained a physiological ratio of 3:1:1 for acetate, propionate, and butyrate [Figure 6A], but in response to the cranberry extract supplementation, the SCFA profile changed. The ratios of propionate and, notably, butyrate increased concurrently [Figure 6A]. This outcome was attributed to a rapid and substantial decrease in acetate concentration during the initial week of treatment (6/6 donors) (*P* < 0.001). Although the rate of decline stabilized, it remained significantly lower compared to the control period throughout the second week as well [Figure 6B]. The effect of the supplementation on acetate production is more pronounced in the ascending colon, but still present to a lesser extent in the transverse colon (5/6 donors) [Figure 6B]. The changes in propionate production were not prominent, but still showed a significant increase in response to the cranberry extract in the transverse colon after two weeks of supplementation (5/6 donors) (*P* < 0.01) [Figure 6B]. During the first week of supplementation in the ascending colon, there was a slight decrease in propionate production. However, by the second week, it returned to the same levels observed during the control period (5/6 donors) [Figure 6B]. Minor SCFA (e.g., branched-chain fatty acids and valerate) mean production was also assessed [Supplementary Figure 11]. Overall SCFA concentrations and ratios per donor were also evaluated and are available in Supplementary Figures 12-15. Finally, Spearman correlation analyses were performed to establish the relationship between bacterial abundance and the three primary SCFA. The significant findings from these correlation studies are illustrated in Figure 6C-F. Correlations were performed in the lumen and mucus environments separately during the first and second weeks of supplementation. During the first week, many genera correlated positively with the production of acetate in the mucus of the transverse colon (*Alistipes*, *Coprococcus*, *Faecalicatena*, *Monoglobus*, *Parabacteroides*) [Figure 6D], while others correlated with propionate in the lumen (*Atlantibacter*, *Citrobacter*, and *Faecalibacterium*) [Figure 6C] and in the mucus (*Aminipila*, *Citrobacter*, and *Pseudoflavonifractor*) [Figure 6D]. Only the *NK4A214 group* from the *Oscillospiraceae* family correlated strongly with butyrate production in the mucus environment [Figure 6D]. After two weeks of supplementation, various other genera demonstrated a correlation with butyrate production. In the lumen, this is the case for *Subdoligranulum* and *Akkermansia* [Figure 6E]. Moreover, in the mucus, *Akkermansia* exhibited a significant correlation alongside other genera such as *Agathobaculum*, *Lactiplantibacillus*, *Lactonifractor*, *Megasphaera*, and *Phascolarctobacterium*, among others, as depicted in Figure 6D. This last genus also correlated with propionate in both environments [Figure 6C and D]. In the ascending colon, several genera correlated with acetate (*Anaerostipes*, *Roseburia*, *Sutterella*), propionate (*Anaerobutyricum*, *Bacteroides*, *Phascolarctobacterium*, *Sutterella*), and butyrate production (*Anaerostipes*, *Lachnoclostridium*, *Roseburia*) [Supplementary Figure 16].

## DISCUSSION

Given the growing body of evidence underscoring the significance of gut microbiota in health and disease^[7,45,46]^, the research emphasis on the health implications of (poly)phenols has evolved. Instead of concentrating on their direct antioxidant properties, current studies are increasingly examining their interactions with the gut microbiota^[8,12-14]^. This is particularly relevant to PAC, since they are poorly absorbed in the upper digestive tract and reach the colon intacts^[7-9]^. This interaction is reciprocal; that is, PAC both alter the composition and are presumed to be metabolized by the gut microbiota. However, this relationship is yet to be fully characterized, and may provide key insight into the crucial mechanism underlying their health benefits. Moreover, the interplay between oligosaccharides, present at approximately 120 mg/day in our study^[28]^, and PAC needed further examination since few studies have taken into account this combination^[10,11]^.

To advance our understanding of the influence of supplementing the microbiota with a cranberry extract, we conducted a two-week study in the *ex vivo* TWIN-M-SHIME®. This system was inoculated with fecal samples from six healthy individuals to ensure an adequate representation of the population and provide a suitable sample size. The TWIN-M-SHIME® enabled us to closely replicate the physiology of the gastrointestinal tract. It not only captured its inherent dynamism, but also provided us the opportunity to investigate the mucosal environment, which is typically inaccessible when conducting *in vivo* human studies.

Although the luminal and mucosal environments have distinct microbiota profiles, it is worth noting that regional variations along the length of the colon are equally significant. These regional differences can influence how nutrients are metabolized^[47]^. The M-SHIME system used in the experiments was set up to replicate the conditions of the ascending and transverse colon, as flavan-3-ols monomers and dimers are typically broken down in these specific regions of the colon^[15]^. Our research conclusively showed that a two-week regimen of cranberry extract supplementation significantly influenced the gut microbiota. The effects of this supplementation varied depending on the particular niche and region within the gastrointestinal tract.

The impact of the supplementation varied notably between the two regions of the colon. In the ascending colon, there was a more pronounced decrease in acetate and a generally more significant modulation of the microbiota. This observation might be attributed to two factors: a rapid metabolism of a fresh batch of unmodified compound upon the transfer of the stomach content directly in the ascending colon, and a dilution impact due to the unique fluid dynamics of the SHIME system^[48]^. The ultimate result of the microbial modulation induced by the cranberry extract was a significant increase in butyrate levels throughout both regions of the colon. This finding aligns with other *in vitro* and *in vivo* studies that have investigated the effects of PAC from various fruits (such as cranberry, aronia berry, and grape) on microbial metabolism, which have also noted an increase in butyrate levels^[15,16,49,50]^. This response is, however, not unique to cranberry (poly)phenols as the ethanolic extract also contains a significant amount of oligosaccharides, which have also been correlated to an increase in the production of butyrate in literature^[11,51]^. In the ascending colon lumen, the supplementation led to an increased relative abundance of several genera, including *Anaerostipes*, *Lactiplantibacillus*, *Phascolarctobacterium*, *Acidaminococcus*, *Clostridium*, and *Roseburia*. These genera are known producers of butyrate^[50]^. In the transverse colon lumen, the butyrate-producing *Anaerostipes*, *Anaerobutyricum*, *Lactiplantibacillus*, *Faecalibacterium*, *Roseburia* and *Subdoligranulum* were stimulated by the supplementation^[50-56]^.

The analysis of co-occurrence networks further revealed the importance of butyrogenic species in the community. Examining co-occurrence patterns and identifying node species can offer more comprehensive insights compared to solely depending on data about species abundance. These approaches can help us infer not only the structure of the microbial community but also its ecological functions and dynamics^[57-60]^. Furthermore, these methods enable the identification of keystone species, which play a pivotal role in the community, irrespective of their abundance^[38,57,60,61]^. Among the species identified as keystones in the lumen of the transverse colon, *Anaerobutyricum soehngenii* has been recently identified as a next-generation probiotic due to its capacity to regulate insulin sensitivity by producing butyrate from acetate and D,L-lactate and influencing bile acid metabolism^[62]^. *Megasphaera micronuciformis* was also identified as a keystone species capable of producing SCFA, including acetate, propionate, butyrate, and, notably, valerate. This species was predominant in the microbiota of only one donor, which also had an elevated production of valerate. Therefore, its importance could be derived from this specific donor data. *F. plautii* emerged as another keystone species. Known for its butyrogenic properties^[63]^, this species has been documented to be involved in the metabolism of catechins^[8]^ and in the attenuation of gut inflammation^[64-66]^. The last keystone of the lumen environment, *B. vulgatus*, has been shown to attenuate gut microbiota lipopolysaccharides production and can utilize succinate to produce propionate^[67,68]^. Propionate plays a role in the regulation of satiety, the reduction of cholesterol and lipogenesis, and the maintenance of metabolic homeostasis^[16,67]^. The production of this SCFA was also increased after supplementation and several other genera were recognized as propionate-producers, such as *Akkermansia*, *Alistipes*, *Anaerobutyricum*, *Phascolarctobacterium*, and *Roseburia*, which were stimulated in the mucus environment of the transverse colon^[50-56]^. In the mucus environment, *A. hallii* also produces butyrate as well as propionate from the same secondary acids or from metabolites of previously degraded complex carbohydrates^[69]^. It has been documented that *A. hallii* engages in cross-feeding by utilizing acetate and lactate generated by lactobacilli and bifidobacteria species in culture^[52,69]^. This cross-feeding interaction has been observed, particularly with *B. adolescentis*^[53]^. Additionally, these *Anaerobutyricum* species also partake in symbiotic relationships with mucin-degrading bacteria^[69,70]^. In particular, *A. hallii* leverages 1,2-propanediol generated by *A. muciniphila* from fucose to synthesize propionate^[70]^. These two bacteria hold great potential as next-generation probiotics. In fact, specific strains are already available on the market as components of a dietary supplement that demonstrated improved post-prandial glucose control in subjects with type 2 diabetes^[71]^. The enhancement of *A. muciniphila* by PAC and (poly)phenol-rich foods has been well documented in scientific literature^[20,22,24,70]^, and associated with a range of beneficial health effects^[23,24,69,72,73]^, including the stimulation of mucus production and the amelioration of gut barrier integrity^[17,23,24]^. The other identified keystones in the mucus environment, *R. faecis*, *I. butyriciproducens*, and *A. butyriciproducens*, are also butyrate-producers^[50,51,74-76]^, while *H. saccharivorans* is a carbohydrate consumer that may contribute to cross-feeding in the ecosystem by producing H_2_ and acetate^[77]^.

The existence of a consortium of butyrate-producing bacteria within the mucus environment strongly suggests a specialized ecological role. The presence of butyrate in this ecological niche is important as it facilitates its delivery near the epithelial layer, where it can enhance the integrity of the barrier^[50,67,78]^. The importance of the mucosal niche is extremely important in the interplay of *Bifidobacteria/Proteobacteria*. The mucus provided a favorable environment for *Bifidobacteria* to thrive during the supplementation, unlike the *Escherichia-Shigella*, which did not proliferate as they had in the lumen. The increased abundance of beneficial bacteria, including *Bifidobacteria* and other butyrate-producing genera like *Lactiplantibacillus*, *Roseburia*, and *Anaerostipes*, is concordant with various indicators suggestive of a healthy microbial community associated with mucus. Such a microbiota composition could potentially blunt intestinal inflammation^[79-82]^. Conversely, several other genera, such as *Bifidobacteria*, could not withstand the elevated PAC concentration in the lumen of the ascending colon, leading to a substantial decline. This is consistent with other *ex vivo* studies using the SHIME system with various other (poly)phenols supplementations such as grape seed, red wine and black tea, aronia berry, and pomegranate^[83]^. However, in the mucus, *Bifidobacteria* managed to not only survive but also prosper. Such a dual relationship has been documented in past studies, where *Bifidobacteria* succeeded in establishing a niche within the mucus^[84-86]^. *B. adolescentis* emerged as the dominant *Bifidobacteria* species across the fermentation system and responded positively to the supplementation, particularly in the mucus environment. This result was also recently confirmed in a human clinical trial conducted by our group, where a bloom of *B. adolescentis* was observed in participants who consumed the same supplementation regimen as in the present experiment^[28]^. Research has demonstrated that several strains can adhere to the epithelium and metabolize host mucin glycans^[50,87-89]^, in line with our observed colonization of the mucus. The favorable reaction of *B. adolescentis* to the supplementation could also be linked to oligosaccharides contained in the cranberry extract. *Bifidobacteria* are capable of metabolizing this particular carbon source for growth^[50,87,88]^. Recent research has revealed multiple health benefits attributed to *B. adolescentis*, including regulation of inflammation, immune responses^[87,89-92]^, and alleviating chronic metabolic disorders^[92,93]^. *B. adolescentis* breaks down non-digestible carbohydrates or mucin glycoproteins to produce acetate and lactate. These compounds then serve as substrates for butyrate-producing strains like *F. prausnitzii*, *A. hallii*, *A. caccae*, or *Roseburia* sp.^[52-55]^, all of which showed an increase in response to our PAC supplementation. Ecologically speaking, this underscores the significance of cross-feeding among the diverse inhabitants of the mucosal ecological niche, leading to the ultimate production of butyrate and its ensuing physiological impacts.

Interestingly, the combined effect of PAC and oligosaccharides may be more impactful than either component alone. The binding of (poly)phenols to carbohydrates not only enhances their solubility, making it easier for them to reach the colon^[10,56]^, but also uniquely influences the composition of the gut microbiota through their simultaneous metabolism^[11]^. For example, it has been demonstrated that *L. plantarum* can grow on oligosaccharides alone, but utilizes them more efficiently in the presence of PAC^[56]^. Hence, the significant increase in *L. plantarum*, another species known for its butyrate-producing abilities^[94,95]^ observed in all colon compartments and environments in our study, could potentially be attributed to this synergistic effect between PAC and oligosaccharides.

*L. plantarum* is also one of the species involved in PAC metabolism. Two other species known to be active in this metabolic process were stimulated by the supplementation, but only in the transverse colon. *Eggerthella lenta* in the mucus environment is capable of converting (+)catechin and (-)epicatechin into 1-(3’,4’-dihydroxyphenyl)-3-(2”,4”,6”-trihydroxyphenyl)propan-2-ol^[8]^. *F. plautii*, present in both the mucus and the lumen, can further convert 1-(3’,4’-dihydroxyphenyl)-3-(2”,4”,6”-trihydroxyphenyl)propan-2-ol into 5-(3’,4’-dihydroxyphenyl)-γ-valerolactone and 4-hydroxy-5-(3’,4’-dihydroxyphenyl)valeric acid^[8]^. Flavan-3-ols metabolites phenyl-γ-valerolactones and phenylvaleric acids, as confirmed by a metabolomic analysis [Supplementary Figure 17], were exclusively detected in the transverse colon compartments, consistent with previous findings^[96]^.

The presence of PAC-degrading species is one example of inter-individual variability observed in the present study: *E. lenta* was stimulated only in one donor out of six. We observed several differences between the six subjects, not only in the baseline microbiota but also in response to the PAC supplementation. Notably, distinct SCFA-producing species have been stimulated depending on the donors, such as *Akkermansia*, *Alistipes*, *Lactiplantibacillus*, *Phascolarctobacterium*, *Faecalibacterium*, *Subdoligranulum* alongside several members of the *Lachnospiraceae* family. Nevertheless, these differences did not impact the final biological response, as shown by the homogeneous response in SCFA modulation. It is to be anticipated that other biological effects will most probably be different since host-microbiome interaction is a crucial factor to be taken into account.

While this study offers valuable insights into the interaction between cranberry flavan-3-ols, oligosaccharides, and gut microbiota, several limitations should be acknowledged to contextualize the findings and suggest avenues for future research. Firstly, the *ex vivo* TWIN-M-SHIME system, while sophisticated and able to simulate the human gastrointestinal tract, cannot completely replicate the complexity of *in vivo* conditions. The physiological interactions that occur within a living organism are absent in this model. Factors such as the immune system, mucosal barrier function, and host metabolic responses play significant roles in shaping gut microbiota and their metabolic outputs. Additionally, metabolites accumulate in bioreactors due to the lack of dialysis, leading to higher absolute concentrations than physiological levels, which could impact gut microbiota dynamics. Oxygen concentrations, pressure, and REDOX potential in the gut rise from the lumen to the mucus, parameters that cannot be accurately replicated in *ex vivo* systems. This can affect the colonization of the mucus, favoring facultative anaerobes such as species from Actinobacteria or Proteobacteria phyla in the colon mucosa. Moreover, SCFAs can accumulate in the mucus of the microcosms of the M-SHIME system, thereby affecting local pH and favoring more acidophilic species. Thus, while the system provides valuable mechanistic insights, the findings may not fully translate to human physiology. Furthermore, the study involved fecal samples from only six healthy individuals. While this number is adequate for mechanistic analysis in an *ex vivo* system, it limits the generalizability of the results. Inter-individual variability in gut microbiota composition and response to dietary interventions could mean that the findings are not representative of the broader population. Nevertheless, the effects of the treatment on microbiota modulation in our experiment are comparable to those observed in a recent human clinical study on 28 subjects conducted by our group, in which we observed a strong bifidogenic effect that corroborates the *in vitro* model observations and reliability. Finally, although the study provided mechanistic insights into the interactions between cranberry extract and gut microbiota, these mechanisms were not confirmed in the context of host interactions. Incorporating host cells and innate immune responses in future studies would help to confirm and expand upon the potential effects observed in the *ex vivo* model.

Overall, this study provides novel insights into the intricate relationship between cranberry PAC/oligosaccharides, gut microbiota composition, and their potential health benefits. We realized a host-independent study to gain mechanistic insights into the duplibiotic relation between a cranberry extract and the gut microbiota. The findings suggest that supplementing with a cranberry extract has the potential to modulate the gut microbiota in ways that could promote gut health by ameliorating the SCFA production profile and favoring the establishment of butyrogenic guilds, specifically in the mucus environment. Given the overlooked oligosaccharides content in cranberry products, more mechanistic studies are needed to investigate the interplay between these dietary components and the gut microbiota. Additional research, combined with the study of host cells and their associated innate immune cells, could provide confirmation of the potential effects of the intricate microbial metabolome on the intestinal epithelium.

## DECLARATIONS

### Availability of data and materials

The dataset supporting the conclusions of this article is available in the NCBI repository (SRA Bioproject number PRJNA1046640 at https://www.ncbi.nlm.nih.gov/bioproject/PRJNA1046640.

### Copyright

© The Author(s) 2024.
