# Supplementary material for: Supplementation with a cranberry extract favors the establishment of butyrogenic guilds in the human fermentation SHIME system
Source: Microbiome Res Rep. 2024 Jun 14;3(3):34. doi: 10.20517/mrr.2024.17 (PMC11480733; doi:10.20517/mrr.2024.17)
Supplement: Supplementary file 1 [file mrr-3-3-34-SupplementaryMaterials.pdf]

Supplementary Materials

Supplementation with a cranberry extract favors the establishment of butyrogenic guilds in the human fermentation SHIME system

Valentina Cattero[1,2], Charlène Roussel[1,2,3], Jacob Lessard-Lord[1,2], Denis Roy[1,2], Yves Desjardins[1,2]

[1]Institute of Nutrition and Functional Foods (INAF), Faculty of Agriculture and Food Sciences, Laval University, Quebec City G1V 0A6, Quebec, Canada.
[2]Centre Nutrition, Santé et Société (NUTRISS), INAF Laval University, Quebec City G1V 0A6, Quebec, Canada.
[3]Excellence Research Chair on the Microbiome-Endocannabinoidome Axis in Metabolic Health, Laval University, Quebec City G1V 0A6, Quebec, Canada.

Correspondence to: Valentina Cattero, Prof. Yves Desjardins, Institute of Nutrition and Functional Foods (INAF), Faculty of Agriculture and Food Sciences, Laval University, 2440 Bd Hochelaga Suite 1710, Quebec City G1V 0A6, Quebec, Canada. E-mail: valentina.cattero.1@ulaval.ca; Yves.Desjardins@fsaa.ulaval.ca

Supplementary Table 1. Characterisation of the six donors inoculating the three SHIME experiences

CODE	SHIME	Age	Sex	Nationality	Diet
1	1	24	M	French	Western diet
2	1	28	F	French	Mediterranean diet
3	2	26	F	Italian	Pescetarian diet
4	2	35	M	Canadian	Western diet
5	3	34	F	Mexican	Flexitarian diet
6	3	32	F	Canadian	Western diet

Supplementary Table 2. Topological properties of microbial co-occurrence networks after Cytoscape® analysis

Network	N. of samples	N. of taxa	N. of nodes	N. of edges (+/-)	Avg. n. of neighbors	Clust. coeff.	avgK	avgG	avgC
Lumen Ascending Colon Control week	36	34	82	148	3.160	0.192	3.61	0.04	0.24
Lumen Ascending Colon Supplementation week 1	36	24	110	200	3.636	0.159	3.64	0.03	0.24
Lumen Ascending Colon Supplementation week 2	36	22	94	123	2.705	0.176	2.62	0.09	0.19
Mucus Ascending Colon Control week	24	9	53	47	1.889	0	1.77	0.19	0.44
Mucus Ascending Colon Supplementation week 1	16	6	35	27	2.923	0	1.54	0.16	0.59
Mucus Ascending Colon Supplementation week 2	24	8	83	106	2.364	0.083	2.56	0.11	0.19
Lumen Transverse Colon Control week	36	124	124	293	4.726	0.153	4.73	0.02	0.28
Lumen Transverse Colon Supplementation week 1	36	97	162	386	4.765	0.150	4.77	0.02	0.27
Lumen Transverse Colon Supplementation week 2	36	99	164	459	5.598	0.152	5.60	0.02	0.29

Mucus Transverse Colon Control week	24	43	105	193	3.676	0.144	3.68	0.03	0.24
Mucus Transverse Colon Supplementation week 1	16	55	132	187	2.862	0.097	2.83	0.04	0.18
Mucus Transverse Colon Supplementation week 2	24	62	185	479	5.178	0.149	5.29	0.02	0.27

avgK refers to average degree, avgG to average betweenness centrality, and avgC to average closeness centrality.

Supplementary Table 3. Topological properties of identified keystones species in respective niche of the transverse colon after Cytoscape® analysis

Keystone species (ASV)	Niche	ASV	Degree (K)	Betweenness centrality (G)	Closeness centrality (C)
Megasphaera micronuciformis	Lumen	(ASV_6)	8	0.012	0.315
Bacteroides vulgatus	Lumen	(ASV_25)	7	0.012	0.314
Flavonifractor plautii	Lumen	(ASV_40)	8	0.012	0.315
Anaerobutyricum soehngenii	Lumen	(ASV_68)	7	0.013	0.304
Akkermansia muciniphila	Mucus	(ASV_18)	8	0.002	0.302
Roseburia faecis	Mucus	(ASV_111)	6	0.005	0.265
Intestinimonas butyriciproducens	Mucus	(ASV_205)	6	0.007	0.271
Anaerobutyricum hallii	Mucus	(ASV_231)	6	0.007	0.294
Agathobaculum butyriciproducens	Mucus	(ASV_1052)	6	0.006	0.265
Hydrogenoanaerobacterium saccharovorans	Mucus	(ASV_1096)	7	0.006	0.268

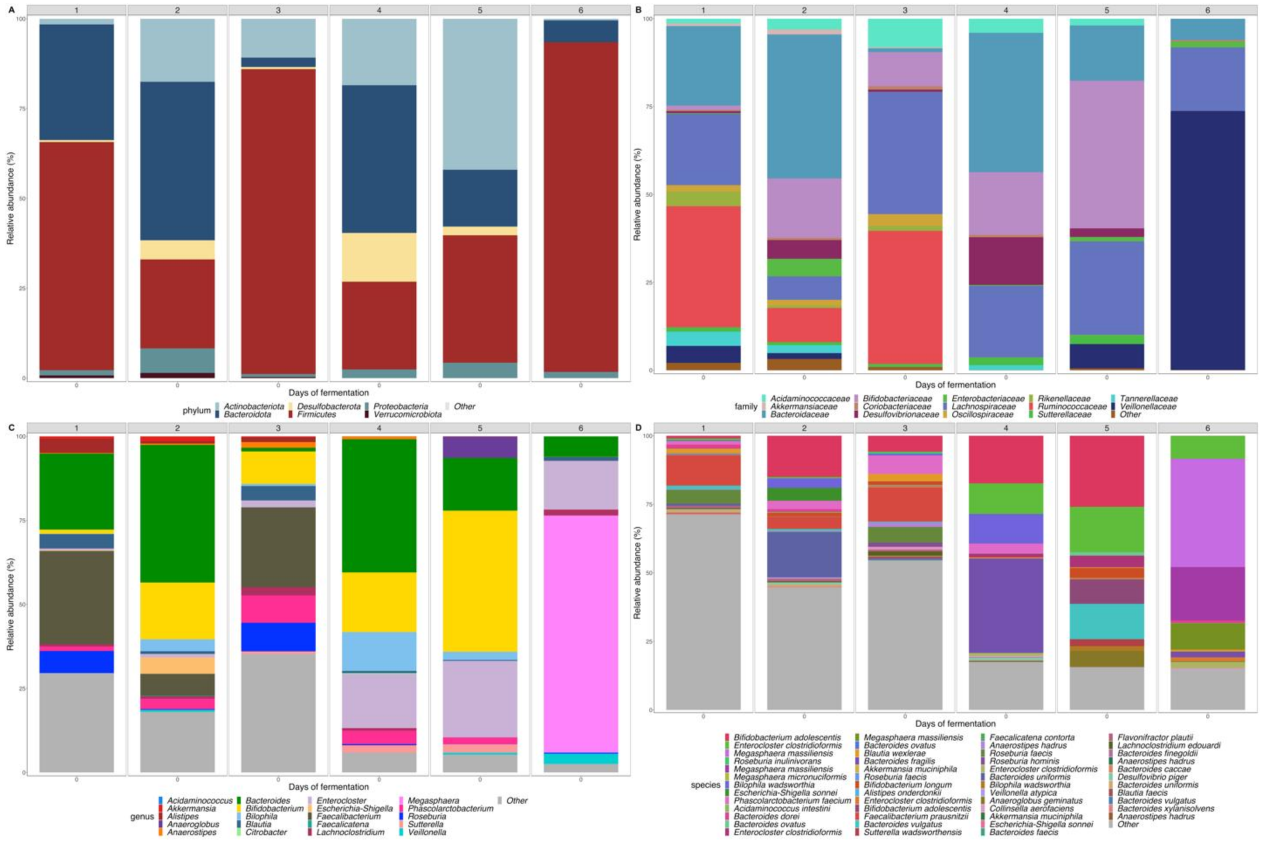

Supplementary Figure 1. Individual microbiota composition of the six donors' faecal inoculum. Stacked barplots of gut microbiota phylum (A), family (B), genus (C) and ASV (D) level relative abundance of the faecal inoculum used to inoculate the SHIME system for the six donors.

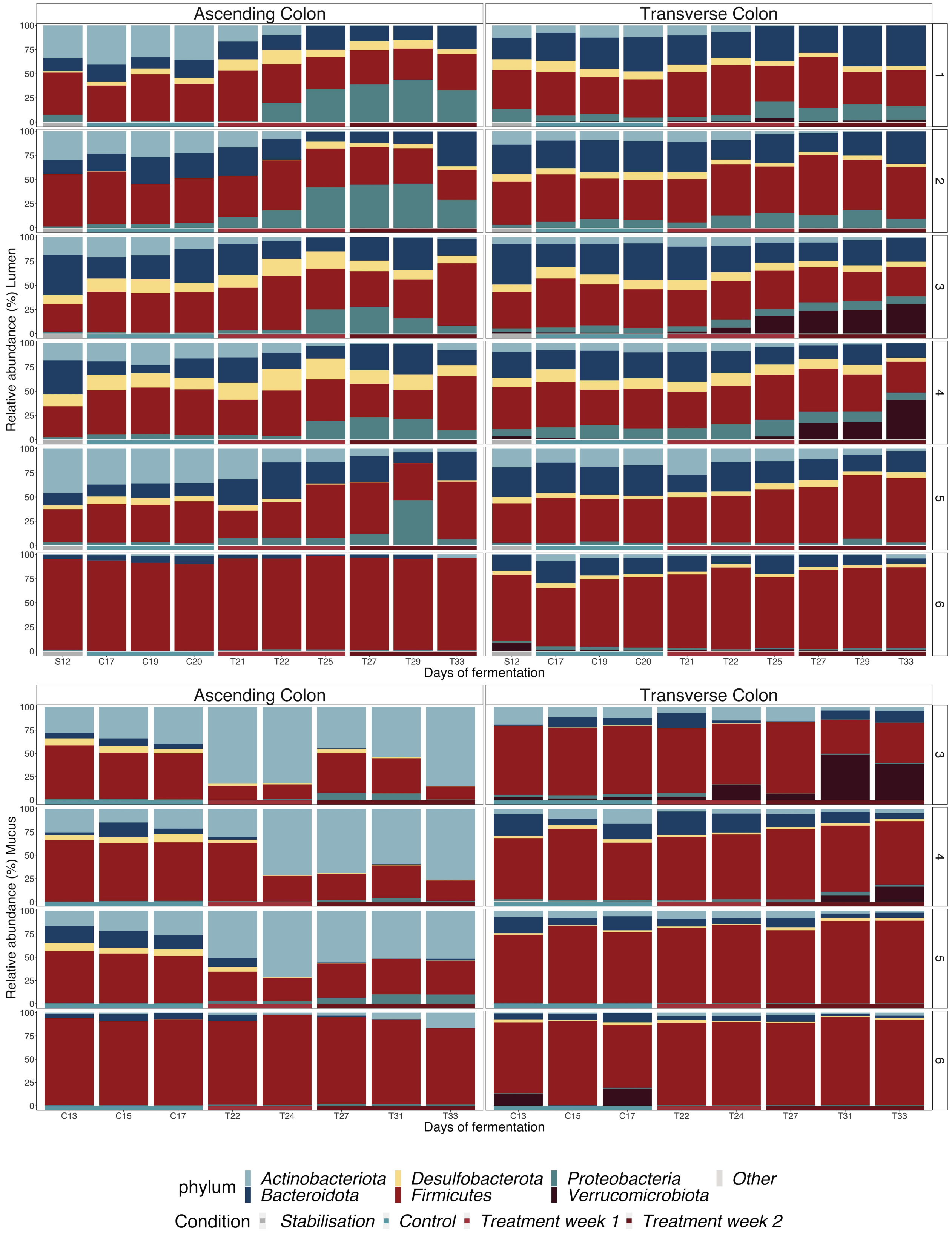
Ascending Colon
Transverse Colon
Relative abundance (%) Lumen
Days of fermentation
S12 C17 C19 C20 T21 T22 T25 T27 T29 T33
1
2
3
4
5
6
Relative abundance (%) Mucus
C13 C15 C17 T22 T24 T27 T31 T33
phylum
Actinobacteriota
Bacteroidota
Desulfobacterota
Firmicutes
Proteobacteria
Verrucomicrobiota
Other
Condition
Stabilisation
Control
Treatment week 1
Treatment week 2

Supplementary Figure 2. Individual microbiota composition of the six donors during the fermentation period at the phylum level. Stacked barplots of gut microbiota phylum level relative abundance during specified days of fermentation for the six donors separately: following two weeks of stabilization and one week of control period, the extract was added from the 21st day, at 86,8mg/PAC/day/donor. The graphic indicates the one-week control period (C; blue line), the first week of cranberry treatment (T; light red line) and the second week of cranberry treatment (T; dark red line). The top graph shows the proportion of the 6 most abundant phyla in the lumen, while the bottom one of the mucus in the respective ascending and transverse colon.

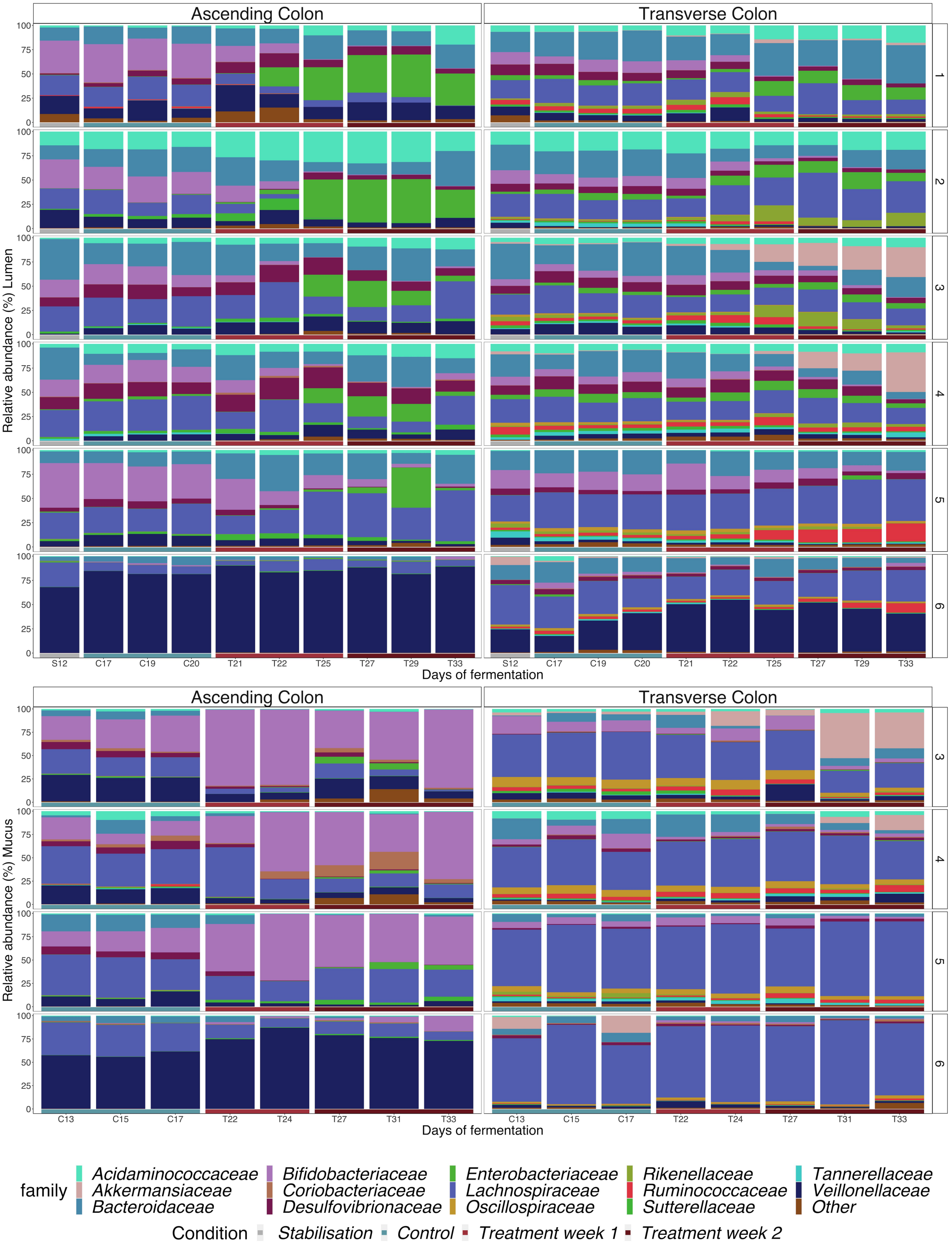

Ascending Colon
Transverse Colon
Relative abundance (%) Lumen
Days of fermentation
S12
C17
C19
C20
T21
T22
T25
T27
T29
T33
1
2
3
4
5
6
Relative abundance (%) Mucus
C13
C15
C17
T22
T24
T27
T31
T33
family
Acidaminococcaceae
Akkermansiaceae
Bacteroidaceae
Bifidobacteriaceae
Coriobacteriaceae
Desulfovibrionaceae
Enterobacteriaceae
Lachnospiraceae
Oscillospiraceae
Rikenellaceae
Ruminococcaceae
Sutterellaceae
Tannerellaceae
Veillonellaceae
Other
Condition
Stabilisation
Control
Treatment week 1
Treatment week 2

Supplementary Figure 3. Individual microbiota composition of the six donors during the fermentation period at the family level. Stacked barplots of gut microbiota family level relative abundance during specified days of fermentation for the six donors separately: following two weeks of stabilization and one week of control period, the extract was added from the 21st day, at 86,8mg/PAC/day/donor. The graphic indicates the one-week control period (C; blue line), the first week of cranberry treatment (T; light red line) and the second week of cranberry treatment (T; dark red line). The top graph shows the proportion of the 15 most abundant families in the lumen, while the bottom one of the mucus in the respective ascending and transverse colon.

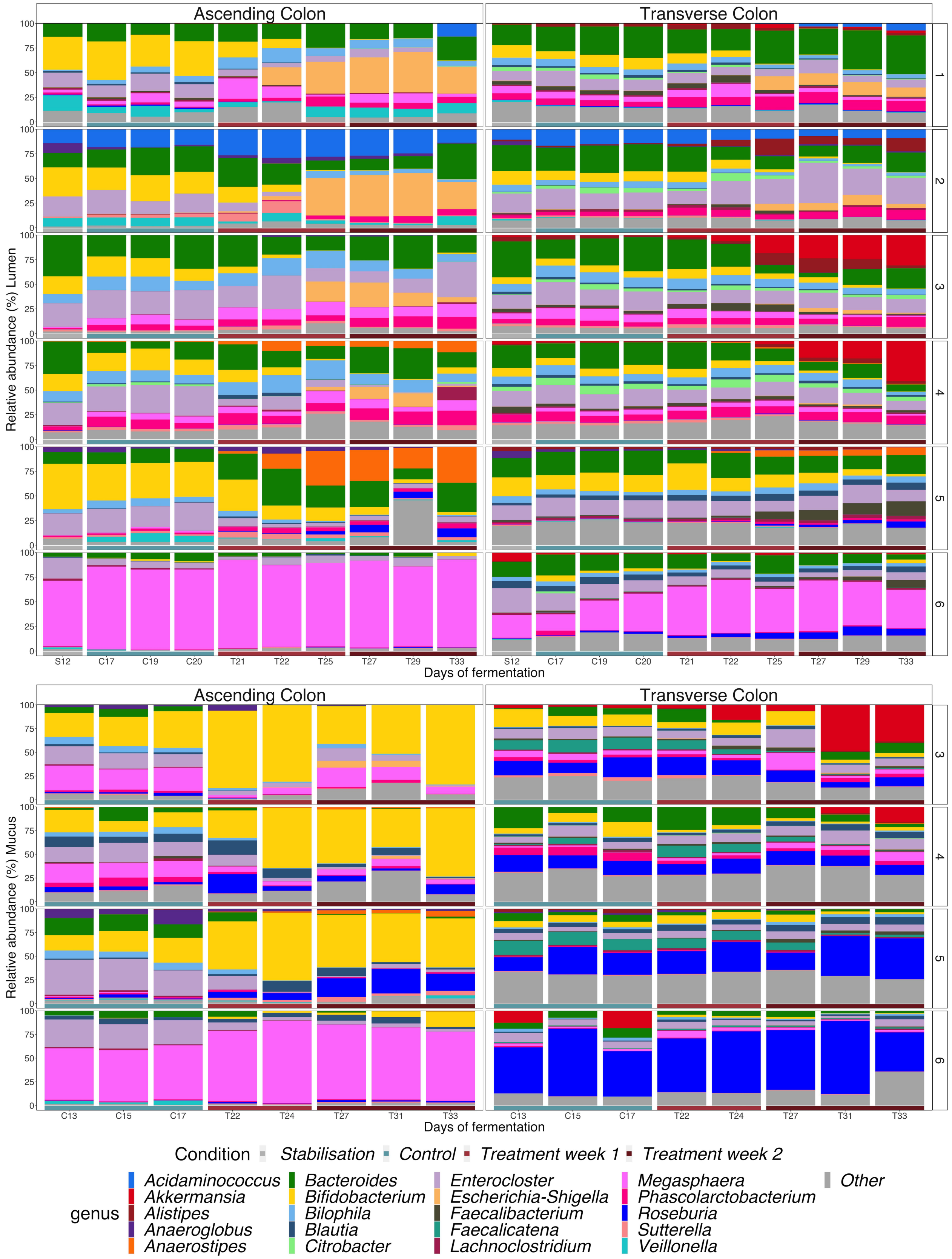
Ascending Colon
Transverse Colon
Relative abundance (%) Lumen
Relative abundance (%) Mucus
Days of fermentation
S12 C17 C19 C20 T21 T22 T25 T27 T29 T33
C13 C15 C17 T22 T24 T27 T31 T33
1
2
3
4
5
6
Condition
Stabilisation
Control
Treatment week 1
Treatment week 2
genus
Acidaminococcus
Akkermansia
Alistipes
Anaeroglobus
Anaerostipes
Bacteroides
Bifidobacterium
Bilophila
Blautia
Citrobacter
Enterocloster
Escherichia-Shigella
Faecalibacterium
Faecalicatena
Lachnoclostridium
Megasphaera
Phascolarctobacterium
Roseburia
Sutterella
Veillonella
Other

Supplementary Figure 4. Individual microbiota composition of the six donors during the fermentation period at the genus level. Stacked barplots of gut microbiota genus level relative abundance during specified days of fermentation for the six donors separately: following two weeks of stabilization and one week of control period, the extract was added from the 21st day, at 86,8mg/PAC/day/donor. The graphic indicates the one-week control period (C; blue line), the first week of cranberry treatment (T; light red line) and the second week of cranberry treatment (T; dark red line). The top graph shows the proportion of the 20 most abundant genera in the lumen, while the bottom one of the mucus in the respective ascending and transverse colon.

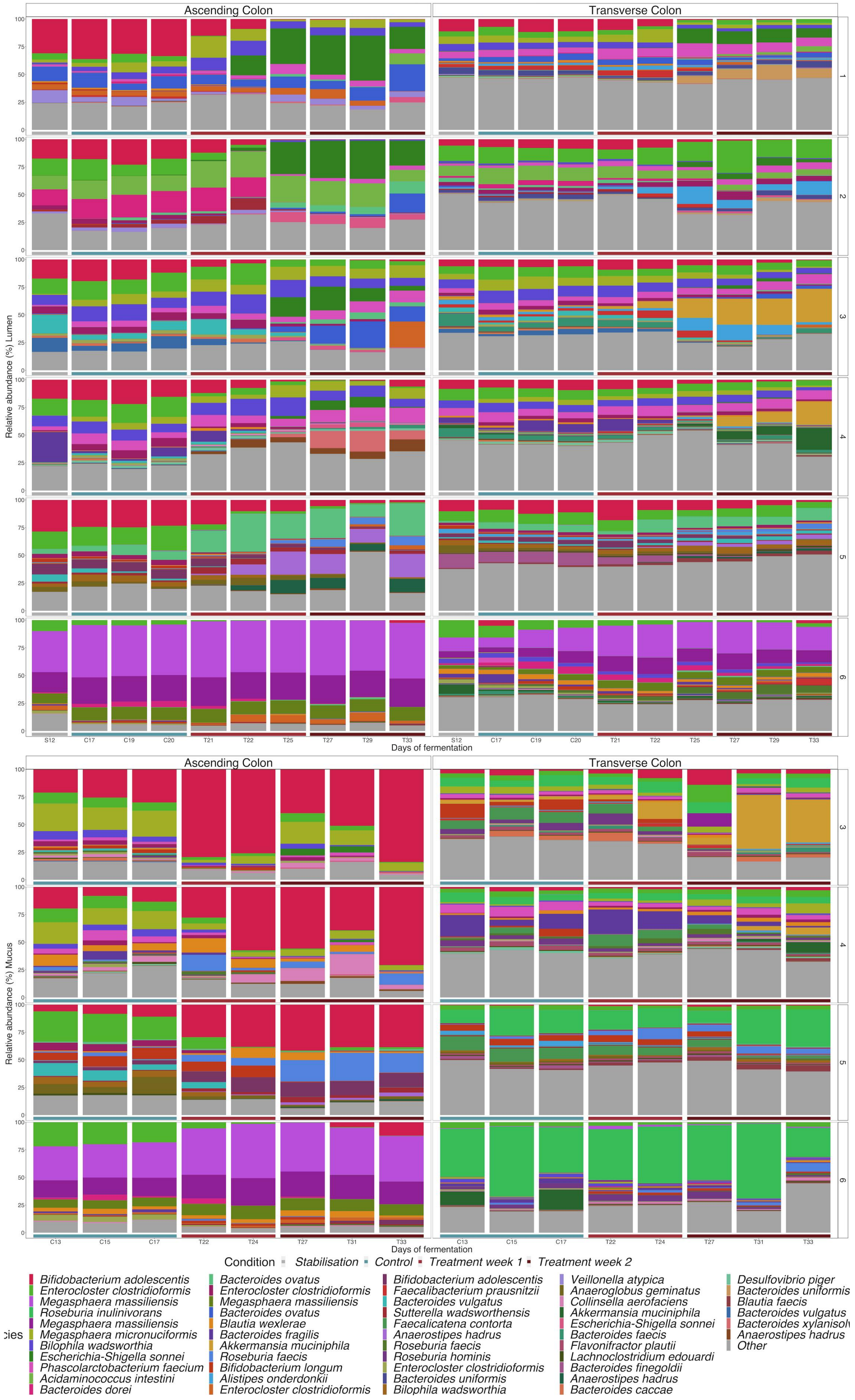

Ascending Colon
Transverse Colon
Relative abundance (%) Lumen
Relative abundance (%) Mucus
100
75
50
25
0
1
2
3
4
5
6
S12 C17 C19 C20 T21 T22 T25 T27 T29 T33
C13 C15 C17 T22 T24 T27 T31 T33
Days of fermentation
Condition Stabilisation Control Treatment week 1 Treatment week 2
cies
Bifidobacterium adolescentis
Enterocloster clostridioformis
Megasphaera massiliensis
Roseburia inulinivorans
Megasphaera massiliensis
Megasphaera micronuciformis
Bilophila wadsworthia
Escherichia-Shigella sonnei
Phascolarctobacterium faecium
Acidaminococcus intestini
Bacteroides dorei
Bacteroides ovatus
Enterocloster clostridioformis
Megasphaera massiliensis
Bacteroides ovatus
Blautia wexlerae
Bacteroides fragilis
Akkermansia muciniphila
Roseburia faecis
Bifidobacterium longum
Alistipes onderdonkii
Enterocloster clostridioformis
Bifidobacterium adolescentis
Faecalibacterium prausnitzii
Bacteroides vulgatus
Sutterella wadsworthensis
Faecalicatena contorta
Anaerostipes hadrus
Roseburia faecis
Roseburia hominis
Enterocloster clostridioformis
Bacteroides uniformis
Bilophila wadsworthia
Veillonella atypica
Anaeroglobus geminatus
Collinsella aerofaciens
Akkermansia muciniphila
Escherichia-Shigella sonnei
Bacteroides faecis
Flavonifractor plautii
Lachnoclostridium edouardi
Bacteroides finegoldii
Anaerostipes hadrus
Bacteroides caccae
Desulfovibrio piger
Bacteroides uniformis
Blautia faecis
Bacteroides vulgatus
Bacteroides xylanisol
Anaerostipes hadrus
Other

Supplementary Figure 5. Individual microbiota composition of the six donors during the fermentation period at the ASV level. Stacked barplots of gut microbiota ASV level relative abundance during specified days of fermentation for the six donors separately: following two weeks of stabilization and one week of control period, the extract was added from the 21st day, at 86,8mg/PAC/day/donor. The graphic indicates the one-week control period (C; blue line), the first week of cranberry treatment (T; light red line) and the second week of cranberry treatment (T; dark red line). The top graph shows the proportion of the 50 most abundant ASV in the lumen, while the bottom one of the mucus in the respective ascending and transverse colon.

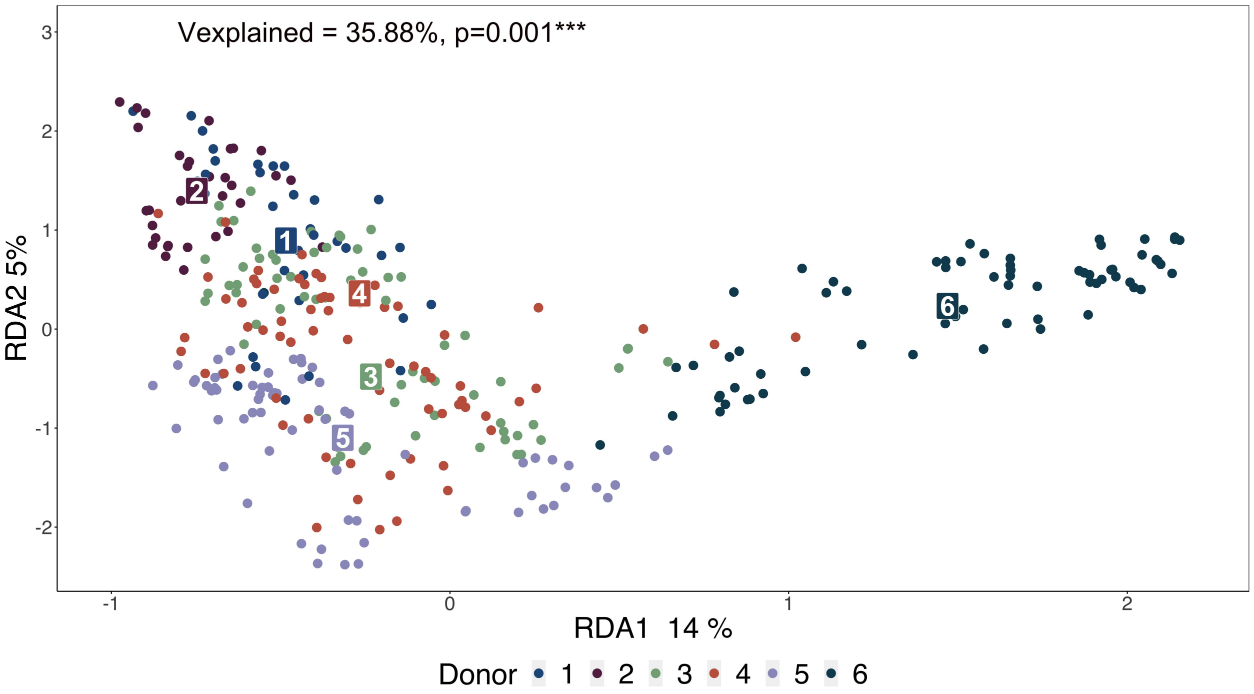

Supplementary Figure 6. Inter-individual variability significantly contributing to the total variation in microbiota composition. Partial distance-based redundancy analysis (db-RDA) of the microbial community composition based on 16S rRNA gene amplicon sequencing showing that inter-individual variability between the six donors (1-6 in different colors) explain the differences in the microbiota. Percent on x and y axes indicate contribution to the total variance, whilst "*Vexplained*" correspond to the variability of the gut microbiota composition explained by the variables (***$P < 0.001$ significance) assessed from the distance matrix PERMANOVA**.**

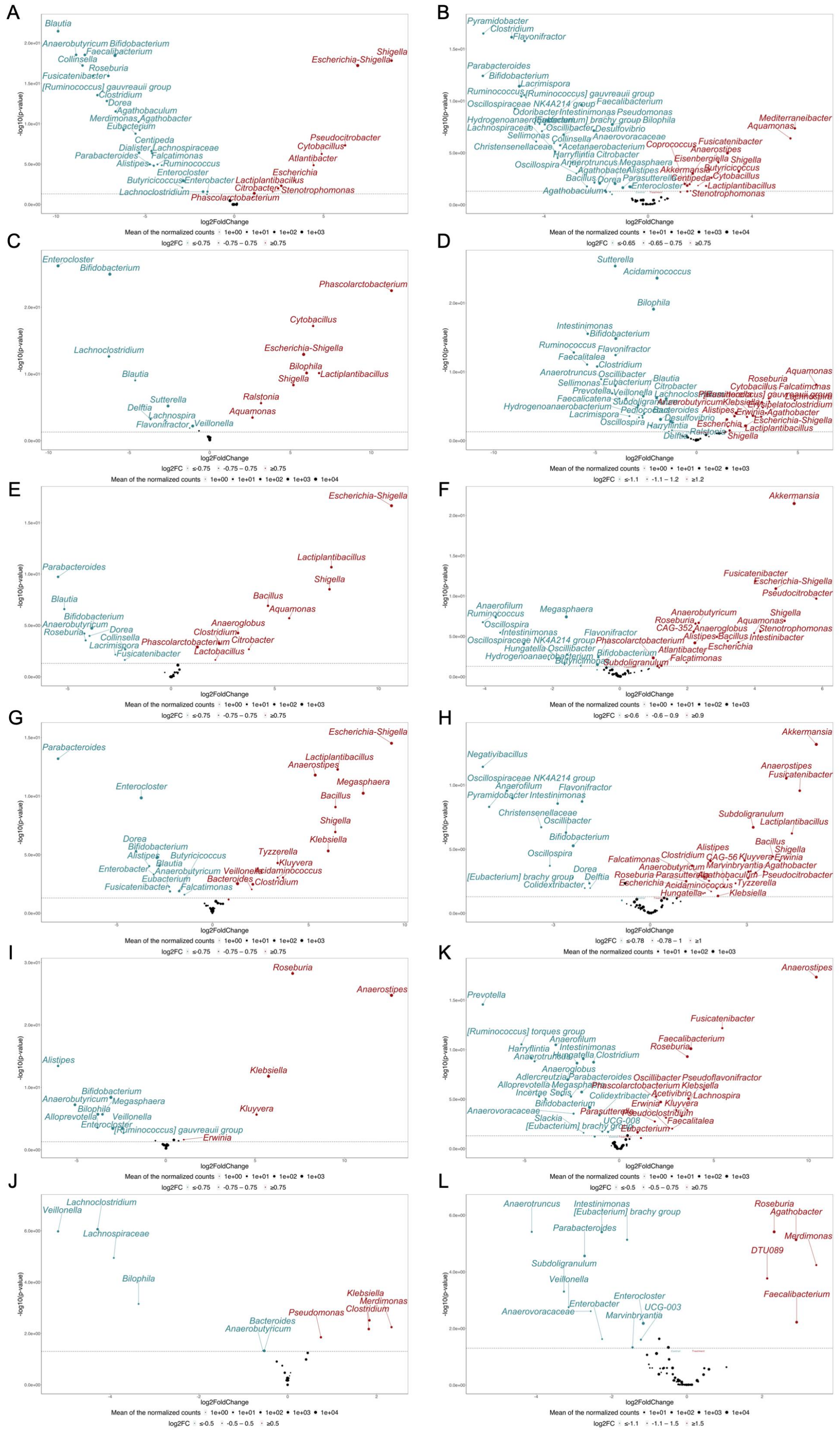
A
B
C
D
E
F
G
H
I
K
J
L
log2FoldChange
-log10(p-value)
Mean of the normalized counts
log2FC

Supplementary Figure 7. Genera enrichment in the lumen of the ascending and transverse colon region of each individual donor. Volcano plots displaying the significantly enriched genera after supplementation as determined using a Wald test with Benjamini-Hochberg multiple testing correction in the lumen of the ascending colon (A, C, E, G, I, J) and of the transverse colon (B, D, F, H, K, L), for each of the six donors. Each line is a different donor: from the top donor 1 (A, B) to the bottom donor 6 (J, L). Deseq2 analysis of log2 fold-changes displays on the left of the x axis (in blue) the genera depleted after the two weeks supplementation, while on the right (in red) the ones enriched after the two weeks supplementation. The y axis shows the log transformed adjusted *P*-value with the dashed line indicating the $\alpha = 0.05$ significance threshold. Statistical differences between the control and the second week of supplementation were determined using a Wald Test with Benjamini-Hochberg multiple correction test.

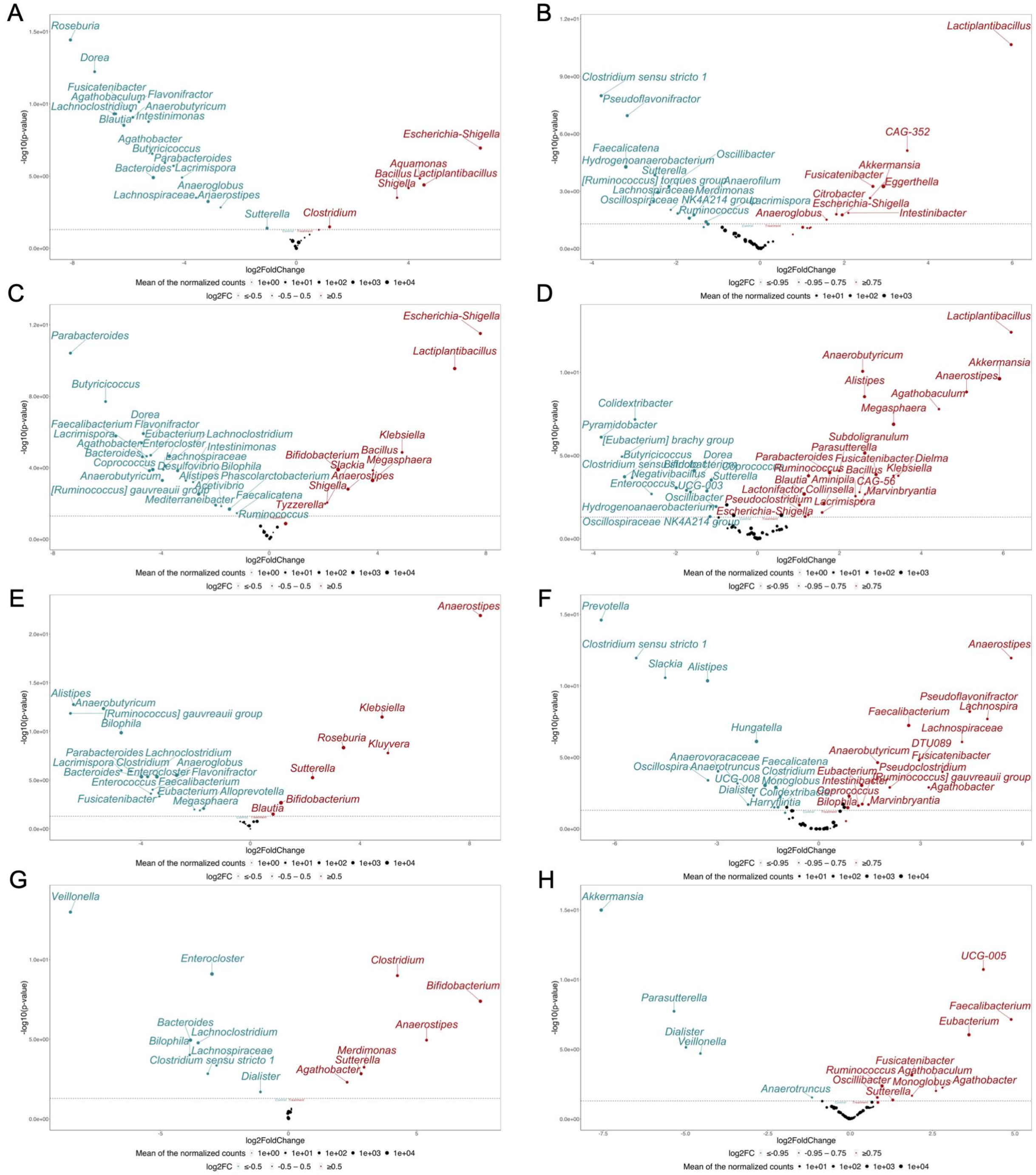

Supplementary Figure 8. Genera enrichment in the mucus of the ascending and transverse colon region of each individual donor. Volcano plots displaying the significantly enriched genera after supplementation as determined using a Wald test with Benjamini-Hochberg multiple testing correction in the mucus of the ascending colon (A, C,E, G) and of the transverse colon (B, D, F,

H), for each of the six donors. Each line is a different donor: from the top donor 1 (A, B) to the bottom donor 6 (G, H). Deseq2 analysis of log2 fold-changes displays on the left of the x axis (in blue) the genera depleted after the two weeks supplementation, while on the right (in red) the ones enriched after the two weeks supplementation. The y axis shows the log transformed adjusted *P*-value with the dashed line indicating the $\alpha = 0.05$ significance threshold. Statistical differences between the control and the second week of supplementation were determined using a Wald Test with Benjamini-Hochberg multiple correction test.

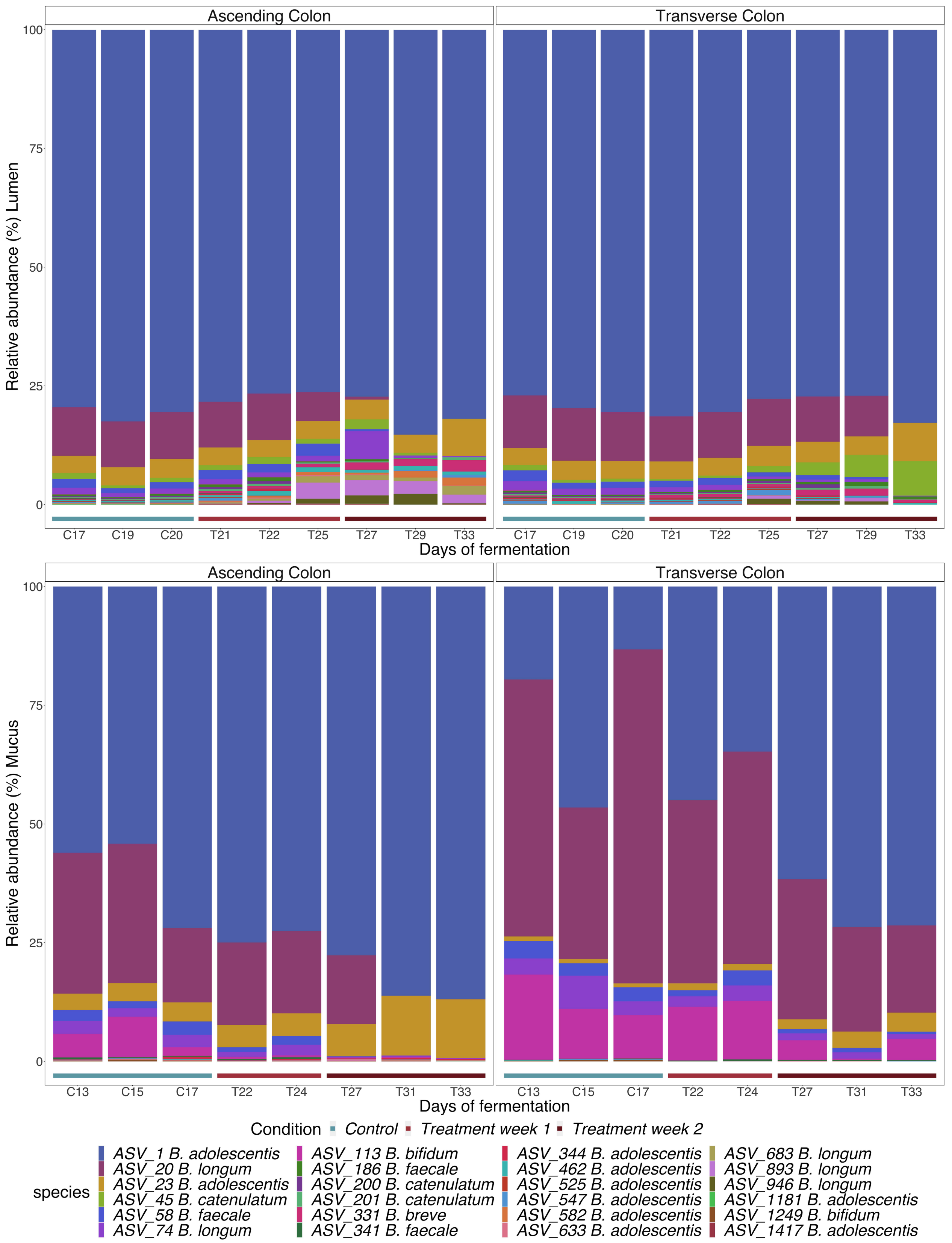
Ascending Colon
Transverse Colon
Relative abundance (%) Lumen
0
25
50
75
100
C17 C19 C20 T21 T22 T25 T27 T29 T33
C17 C19 C20 T21 T22 T25 T27 T29 T33
Days of fermentation
Ascending Colon
Transverse Colon
Relative abundance (%) Mucus
0
25
50
75
100
C13 C15 C17 T22 T24 T27 T31 T33
C13 C15 C17 T22 T24 T27 T31 T33
Days of fermentation
Condition Control Treatment week 1 Treatment week 2
species
ASV_1 B. adolescentis
ASV_20 B. longum
ASV_23 B. adolescentis
ASV_45 B. catenulatum
ASV_58 B. faecale
ASV_74 B. longum
ASV_113 B. bifidum
ASV_186 B. faecale
ASV_200 B. catenulatum
ASV_201 B. catenulatum
ASV_331 B. breve
ASV_341 B. faecale
ASV_344 B. adolescentis
ASV_462 B. adolescentis
ASV_525 B. adolescentis
ASV_547 B. adolescentis
ASV_582 B. adolescentis
ASV_633 B. adolescentis
ASV_683 B. longum
ASV_893 B. longum
ASV_946 B. longum
ASV_1181 B. adolescentis
ASV_1249 B. bifidum
ASV_1417 B. adolescentis

Supplementary Figure 9. Relative abundance of *Bifidobacterium* species (ASVs) during the fermentation period. Stacked barplots of the relative abundance of the ASV associated to the bifidobacteria genus during specified days of fermentation: following two weeks of stabilization and one week of control period, the extract was added from the 21st day, at 86,8mg/PAC/day/donor. The graphic indicates the one-week control period (C; blue line), the first week of cranberry treatment (T; light red line) and the second week of cranberry treatment (T; dark red line). The top graph shows the proportion of the bifidobacteria ASV in the lumen, while the bottom one of the mucus in the respective ascending and transverse colon. The stacked barplots show the mean composition of six donors duplicated. ASV assignation was conducted by NCBI BLASTN and RDP classifier.

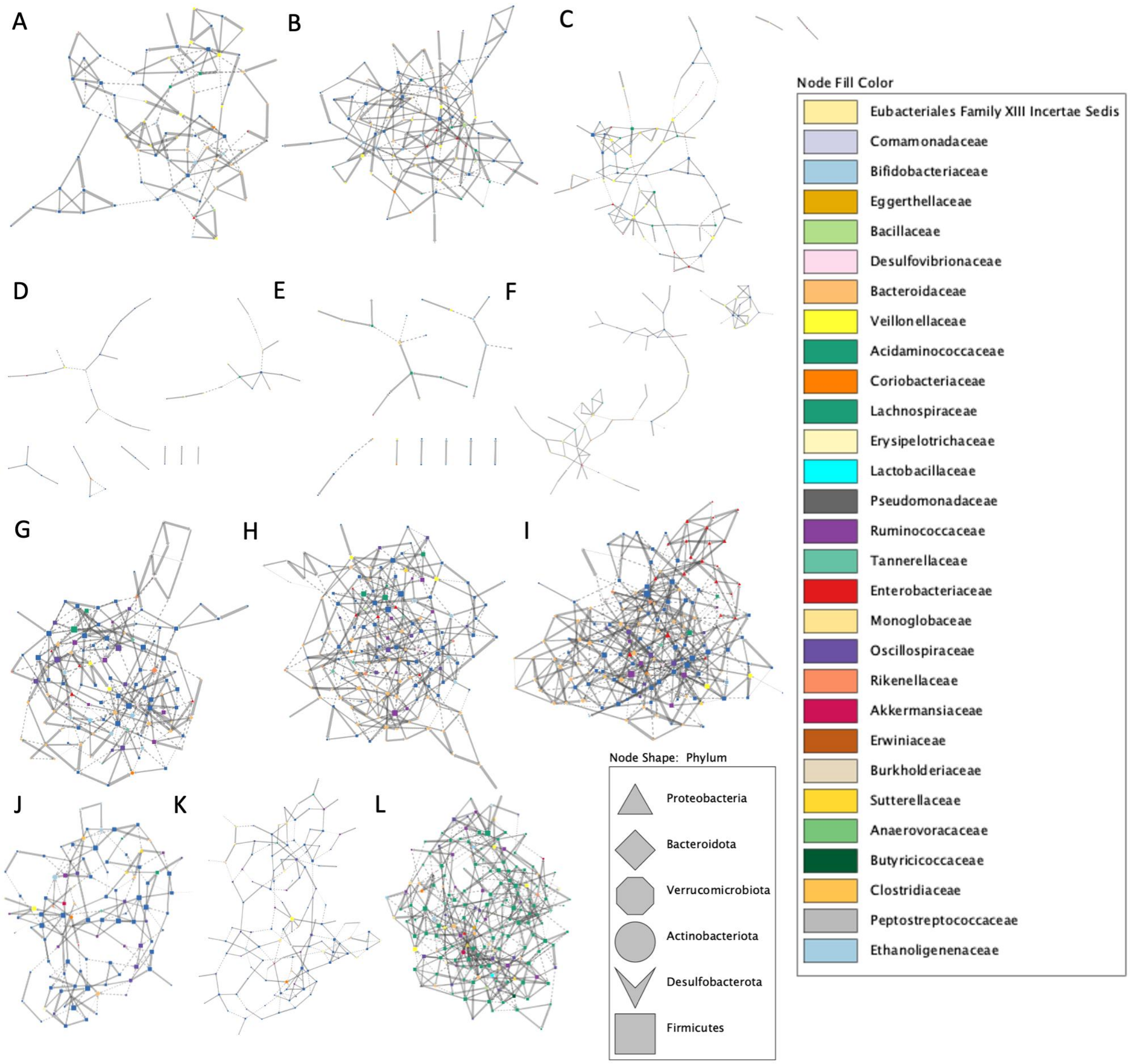

Supplementary Figure 10. Co-occurrence networks during the fermentation period in the TWIN-M-SHIME system across the fermentation. Networks of significant co-occurrences between the bacterial species (ASV) based on 16S rRNA gene amplicon sequencing. (A-C) show the networks for the lumen of the ascending colon during the control, the first and second week of treatment respectively. (D-F) show the networks for the mcuus of the ascending colon during the control, the first and second week of treatment respectively. (G-I) show the networks for the lumen of the transverse colon during the control, the first and second week of treatment respectively. (J-L) show the networks for the mucus of the transverse colon during the control, the first and second week of treatment respectively**.** The nodes are shaped according to the corresponding phylum and colored according to the corresponfing family. The edges indicate a positive (full lines) or negative (dotted lines) correlation between the network nodes.

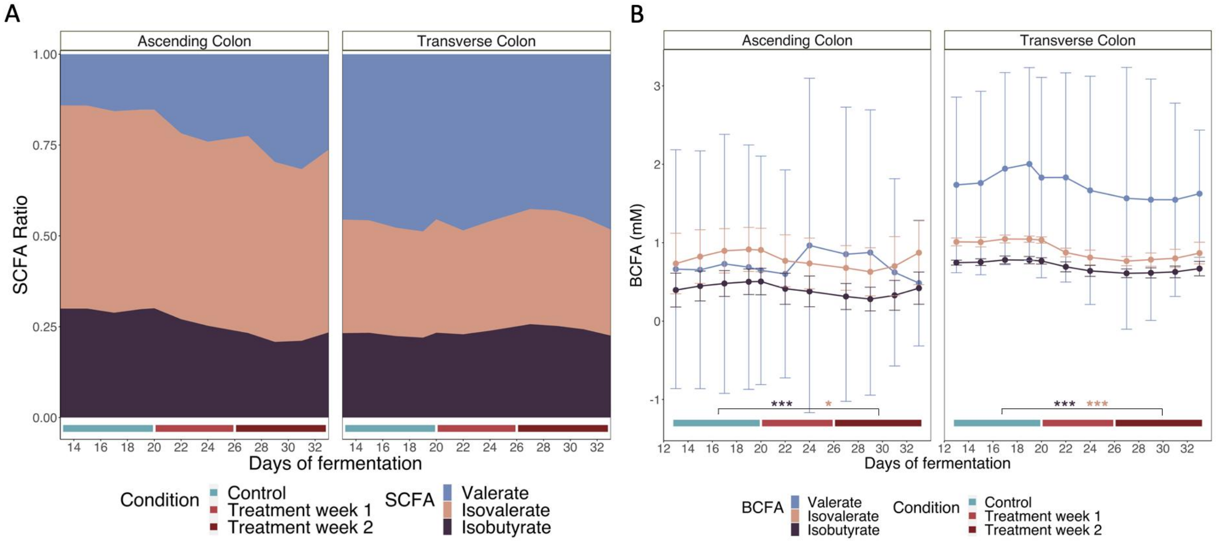

Supplementary Figure 11. Minor SCFA modulation by the cranberry extract supplementation. (A) Mean ratio and (B) mean concentration ±SD over time of the three minor SCFA for the 6 donors in the ascending and transverse colon during the control week (blue line) and the two subsequent weeks of treatment (week 1: light red, week 2: dark red). Statistically significant differences between control and supplementation period are denoted for $P < 0.05$ (*), $P < 0.01$ (**), and $P < 0.001$ (***) as determined by Kruskal-Wallis followed by post-hoc Dunn's test.

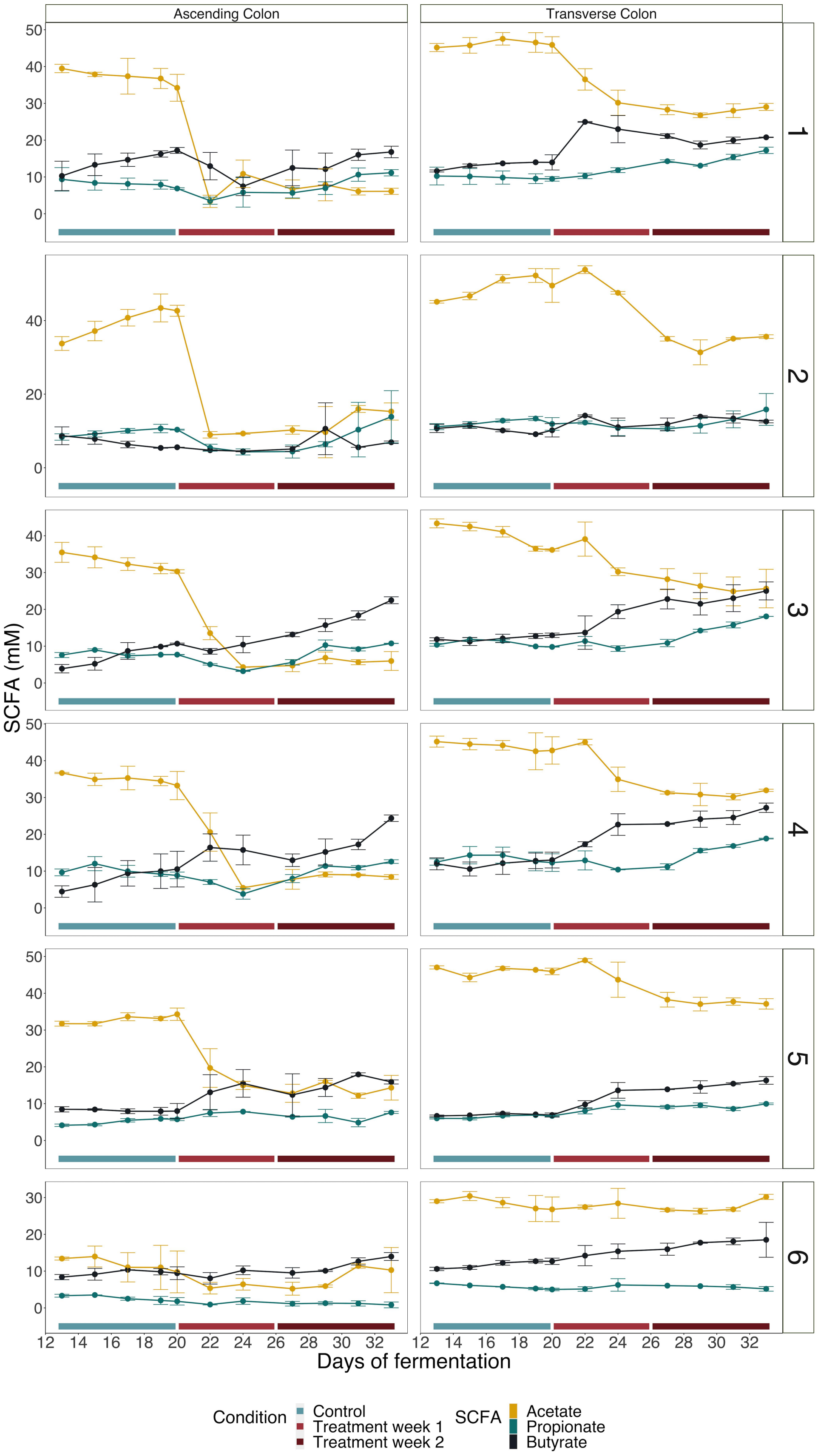

Ascending Colon
Transverse Colon
1
2
3
4
5
6
SCFA (mM)
Days of fermentation
Condition
Control
Treatment week 1
Treatment week 2
SCFA
Acetate
Propionate
Butyrate

Supplementary Figure 12. Major SCFA concentrations per donor during the fermentation period. Mean concentration ±SD over time of the three major SCFA for the 6 donors separately in the ascending and transverse colon during the control week (blue line) and the two subsequent weeks of treatment (week 1: light red, week 2: dark red). Statistically significant differences between control and supplementation period are denoted for $P < 0.05$ (*), $P < 0.01$ (**), and $P < 0.001$ (***) as determined by Kruskal-Wallis followed by post-hoc Dunn's test.

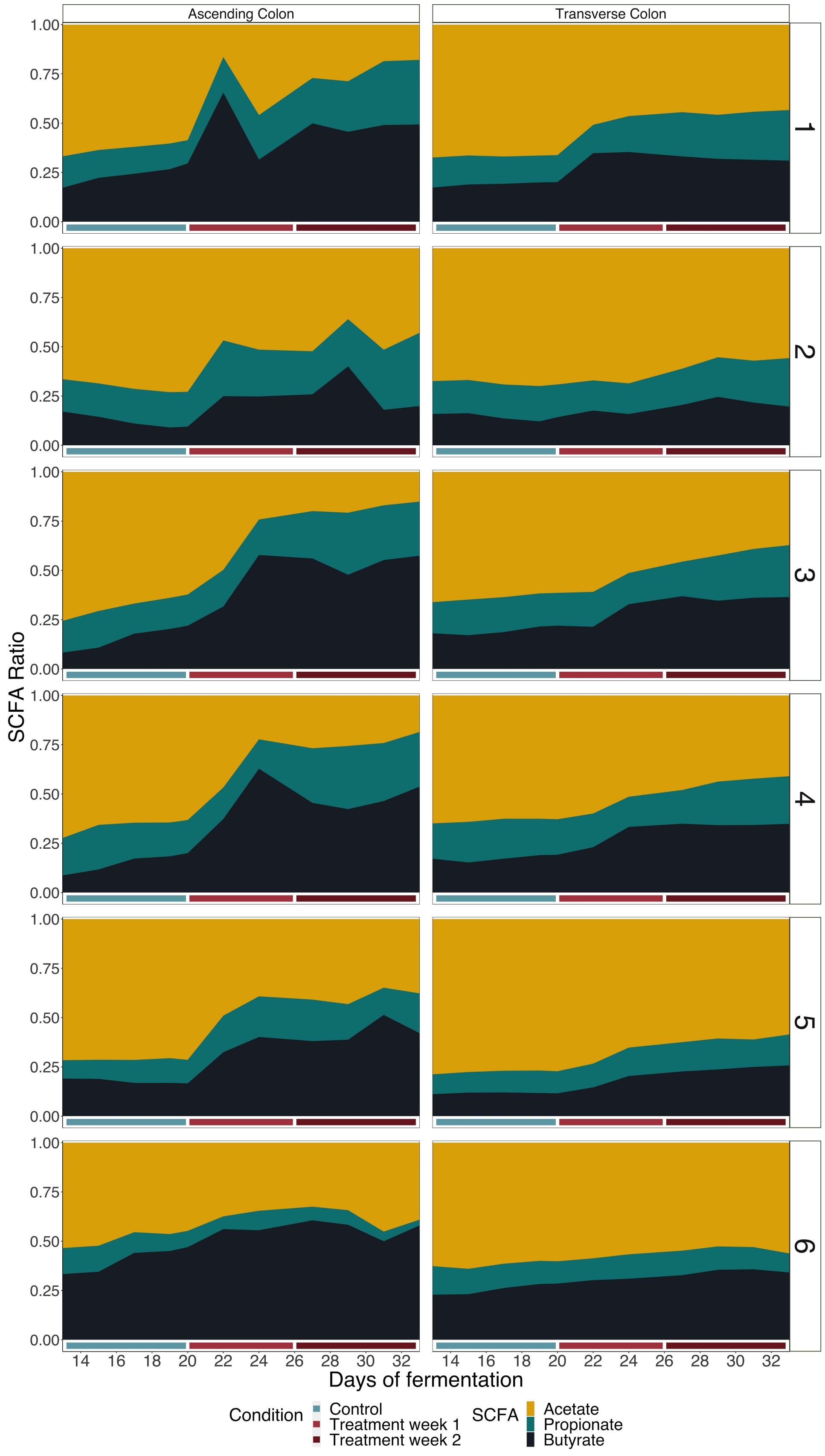

Ascending Colon
Transverse Colon
1
2
3
4
5
6
SCFA Ratio
1.00
0.75
0.50
0.25
0.00
14 16 18 20 22 24 26 28 30 32
Days of fermentation
Condition
Control
Treatment week 1
Treatment week 2
SCFA
Acetate
Propionate
Butyrate

Supplementary Figure 13. Major SCFA ratios per donor during the fermentation period. Mean ratios over time of the three major SCFA for the 6 donors separately in the ascending and transverse colon during the control week (blue line) and the two subsequent weeks of treatment (week 1: light red, week 2: dark red).

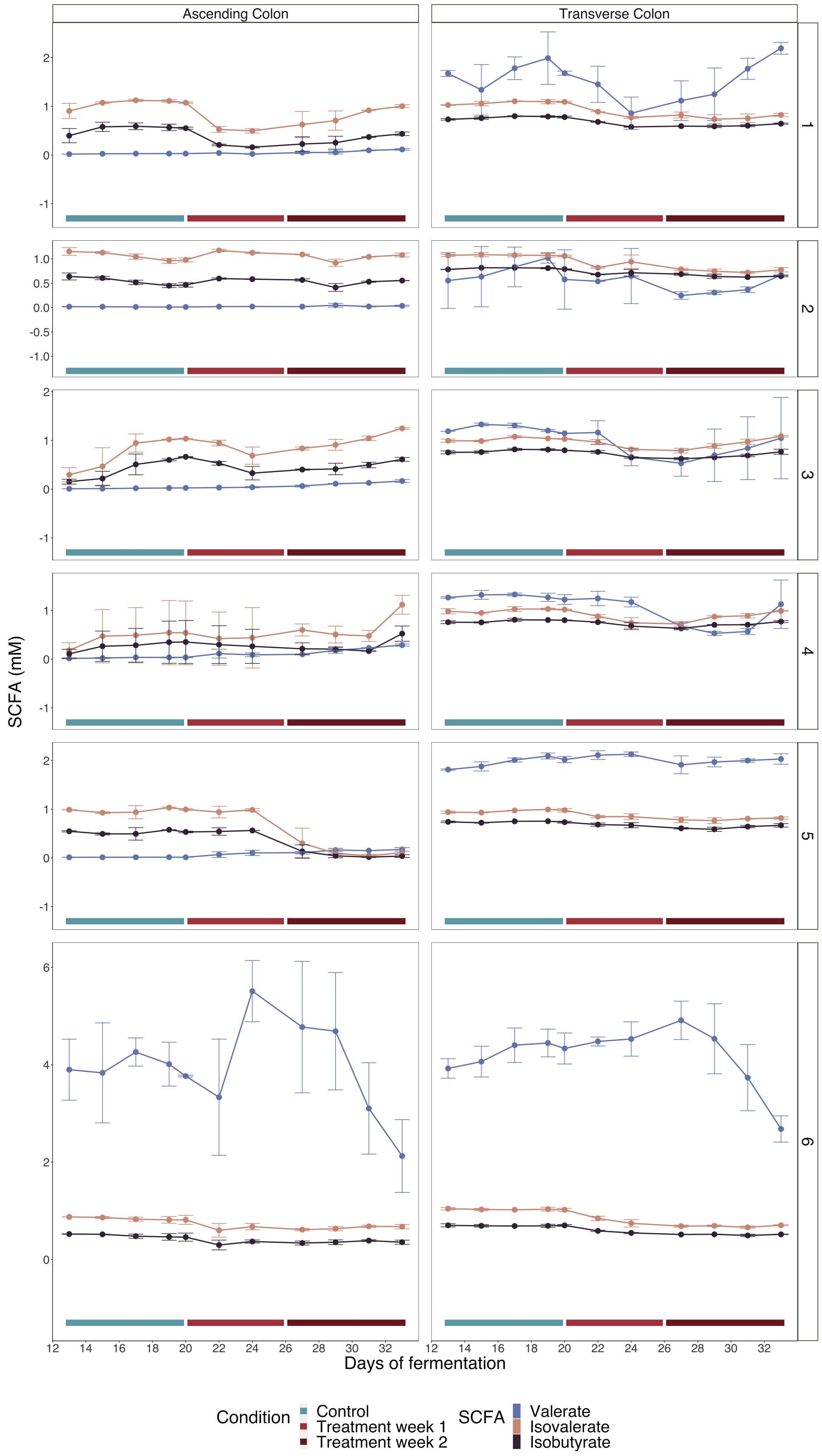

Ascending Colon
Transverse Colon
1
2
3
4
5
6
SCFA (mM)
Days of fermentation
Condition
Control
Treatment week 1
Treatment week 2
SCFA
Valerate
Isovalerate
Isobutyrate

Supplementary Figure 14. Minor SCFA concentrations per donor during the fermentation period. Mean concentration ±SD over time of the three minor SCFA for the 6 donors separately in the ascending and transverse colon during the control week (blue line) and the two subsequent weeks of treatment (week 1: light red, week 2: dark red).

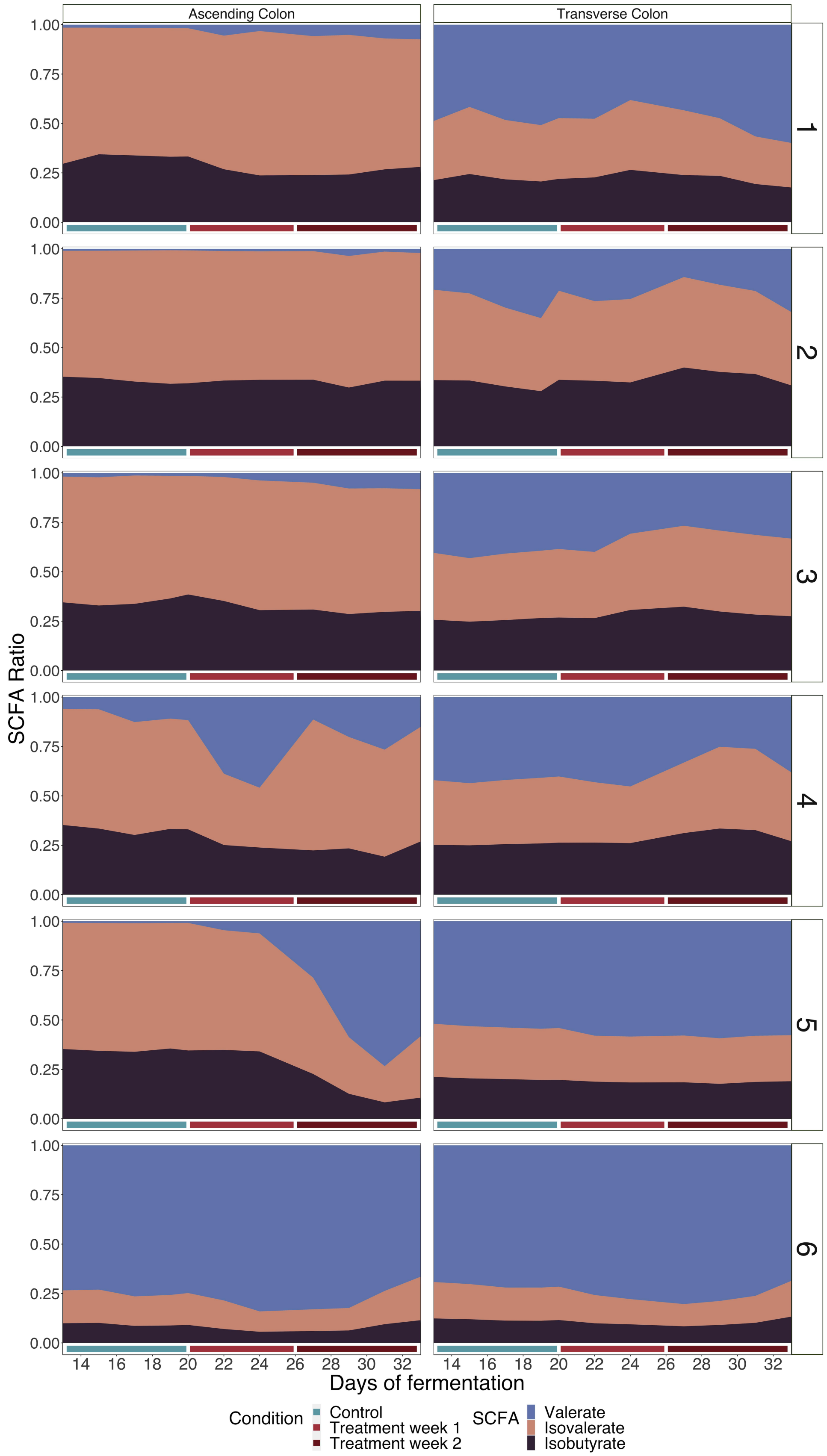

Ascending Colon
Transverse Colon
1
2
3
4
5
6
1.00
0.75
0.50
0.25
0.00
SCFA Ratio
14 16 18 20 22 24 26 28 30 32
Days of fermentation
Condition
Control
Treatment week 1
Treatment week 2
SCFA
Valerate
Isovalerate
Isobutyrate

Supplementary Figure 15. Minor SCFA ratios per donor during the fermentation period. Mean ratios over time of the three minor SCFA for the 6 donors separately in the ascending and transverse colon during the control week (blue line) and the two subsequent weeks of treatment (week 1: light red, week 2: dark red).

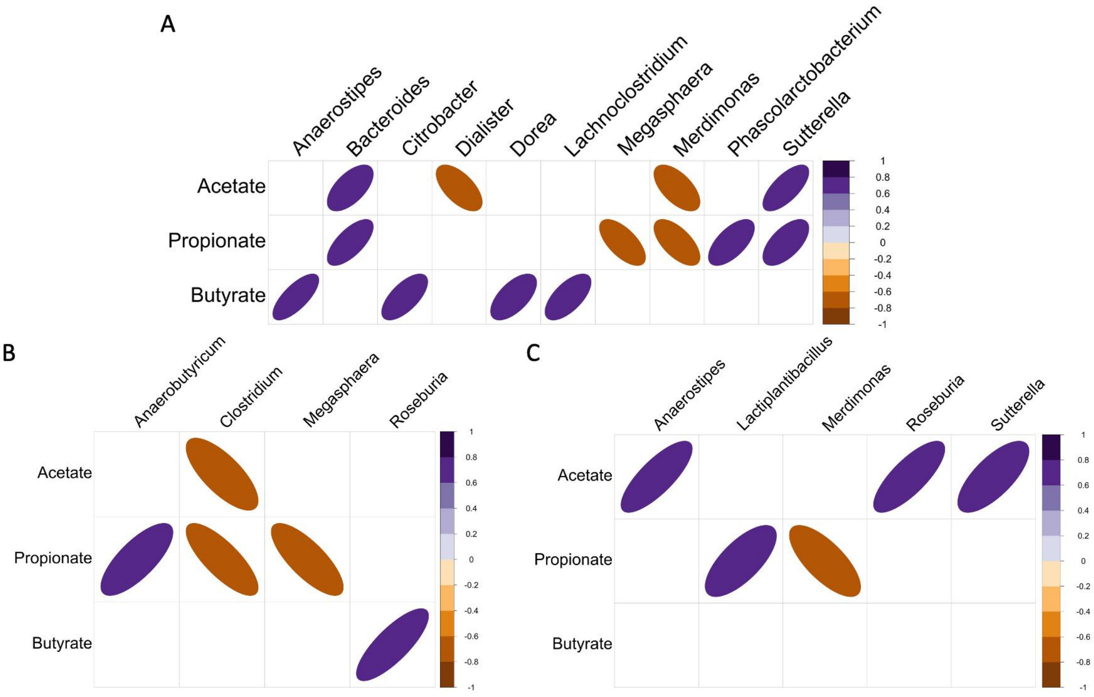

Supplementary Figure 16. Major SCFA correlations with bacterial genera in the ascending colon. Spearman correlations between the three major SCFA and bacterial genera in the ascending colon. Correlations were performed in the lumen (A) and mucus (B and C) environments separately, during the first (B) and second week (A and C) of supplementation. Only negative and positive significant correlations ($P < 0.05$ Benjamini-Hochberg adjusted) that are greater of a 0.7 or -0.7 coefficient are shown, denoted in shades of yellow and purple, respectively.

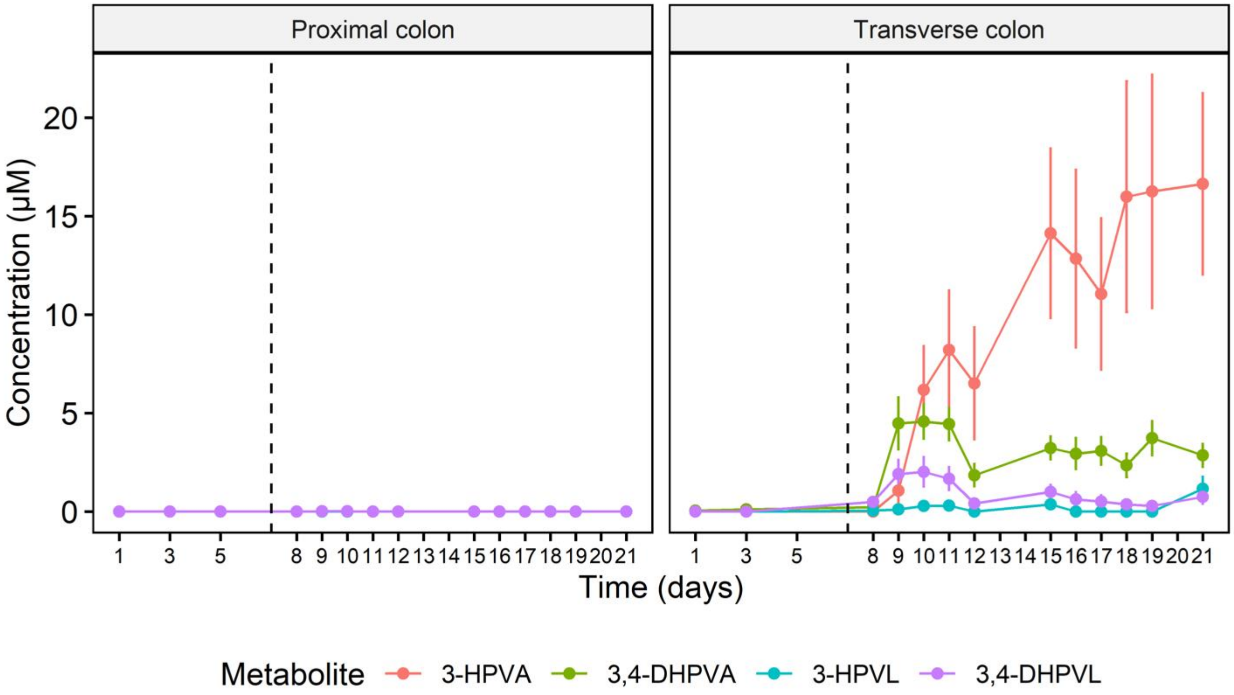

Supplementary Figure 17. Flavan-3-ols metabolites concentrations in the ascending and transverse colon. Concentration (μM) of flavan-3-ols metabolites, namely 5-(3′-hydroxyphenyl)-valeric acid (3-HPVA), 5-(3′,4′-dihydroxyphenyl)valeric acid (3,4-DHPVA), 5-(3′-hydroxyphenyl)-γ-valerolactone (3-HPVL) and 5-(3′,4′-dihydroxyphenyl)-γ-valerolactone (3,4-DHPVL). Concentrations are showed as mean ± standard error for the 6 donors at t=8h in the ascending and transverse colon along the entire fermentation period.

Supplementary Data

TWIN-M-SHIME® system

The first vessel of the SHIME® system was filled three times a day with 140mL of nutritional medium at pH 2 with 45min of residence time to mimic stomach conditions. Subsequently, 60mL of pancreatic/bile juices were added with 1h30 of residence time at pH 6.8 to imitate the transition into the small intestine. The nutritional medium and pancreatic juice composition are detailed in the following table.

1 **Nutritional Medium Composition**

	**g/L**
Arabinogalactan	1.2
Pectin	2
Xylan	0.5
Glucose	0.4
Yeast extract	3
Proteose peptone	1
Mucin	2
L-cystein HCl	0.5
Starch	4

3 **Pancreatic Juice Composition**

	**g/L**
NaHCO3	12.5
Bovine Bile salts (Difco 212820)	6
Pancreatin 4xUSP	0.9

4

2 After this time, the progressive transfer between the vessels took place during 40min. The ascending colon vessels contained 250mL of fecal slurry and the pH was set at a 5.6-5.8 range with a 20h hydraulic residence time. Finally, the transverse colon vessels contained 500mL of fecal slurry and the pH was set at a 6.25-6.4 range with a 32h hydraulic residence time. Details are present in the following table.

TWIN-M-SHIME setup and operation

	Volume	pH	Mucin beads	Pumps	Pump type	1st cycle	2nd cycle	3rd cycle
**NUTRITIONAL MEDIUM x1 (per unit)**	140 ml cycle^{-1} 2x70 ml (420 ml d^{-1})	2	-	Out 4.66 mL min^{-1}	Fast[1] x1	℗ 9-9:30 a.m.	℗ 17-17:30 p.m.	℗ 1-1:30 a.m.
**PANCREATIC JUICE/BILE (PJ) x1**	60 ml cycle^{-1} 2x30 ml (180 ml d^{-1})	6.8	-	Out 4 mL min^{-1}	Fast[1] x1	℗ 10:15-10:30 a.m.	℗ 18:15-18:30 p.m.	℗ 2:15-2:30 a.m.
**STOMACH/ SMALL INTESTINE (ST) x2**	200 ml cycle^{-1} (600 ml d^{-1})	Dynamic 6 to 2 (1h) then 2 to 6.8 (2h)	-	*Residence time*		*9 a.m. – 12 a.m.*	*5 p.m. – 8 p.m.*	*1 a.m. – 4 a.m.*
				Out 4 mL min^{-1}	Slow[2] x2	℗ 12:00 a.m. – 12:39 a.m.	℗ 20:00 p.m. – 20:39 p.m.	℗ 4:00 a.m. – 4:39 a.m.
**ASCENDING COLON (AS) x2**	250 ml	5.6-5.8	2 x 15	*Hydraulic Residence time*		*20h*	*20h*	*20h*
				Out 3 mL min^{-1}	Slow[2] x2	℗ 12:00 a.m. –	℗ 20:00 p.m. –	℗ 4:00 a.m.

						12:54 a.m.	20:54 p.m.	– 4:54 a.m.
**TRANSVERSE COLON (TS) x2**	400 ml	6.25-6.4	2 x 15	*Hydraulic Residence time*		*32h*	*32h*	*32h*
				Out 3 mL min^{-1}	Slow[2] x2	℗ 12:00 a.m. – 12:59 a.m.	℗ 20:00 p.m. – 20:59 p.m.	℗ 4:00 a.m. – 4:59 a.m.

The fecal inoculum for the inoculation of the colonic regions was prepared as a 20% *(w/v)* solution of fresh fecal matter and anaerobic phosphate buffer, as previously described. The bioreactors were then inoculated at 5% *(w/v)* of the fecal inoculum. The microbiotas were fed thrice a day with standard nutritional SHIME medium, that contains floating mucin, as well as bile acids during the whole fermentation period. The microcosms (AnoxKaldnes K1 carrier, Lund, Sweden) coated with type II porcine mucin-agar (Sigma-aldrich, St. Louis, US), which contains MUC2 gel-forming mucin, were used to mimic the mucus-associated microbiotas of the ascending and transverse colons. Samples from the colon vessels were collected every two days, centrifuged, and stored at -20°C for subsequent analysis of short-chain fatty acids (SCFA) and DNA. Samples of mucus-associated microbiota from the microcosms were obtained three times a week. Aliquots of mucus were taken from the colon vessels using a mini sampler spoon (Bel Art™ Scienceware™, Thermo Fisher Scientific, Waltham, US) and stored at -20°C before DNA extraction. The sampling schedule is detailed in the following table:

Sampling schedule during the complete 33-days fermentation period in the TWIN-M-SHIME

	Stabilization				Control							Treatment													
	**0**	**10**	**11**	**12**	**13**	**14**	**15**	**16**	**17**	**18**	**19**	**20**	**21**	**22**	**23**	**24**	**25**	**26**	**27**	**28**	**29**	**30**	**31**	**32**	**33**
**Lumen DNA**	x			x					x		x	x	x	x			x		x		x				x
**Beads DNA**				x	x		x		x					x		x			x				x		x
**SCFA**	x	x	x	x		x		x		x		x		x		x			x		x		x		x
**Metabolomic**	x				x		x		x			x	x	x	x	x			x	x	x	x	x		x
**Organoids**									x			x	x		x	x			x						x

DNA extractions

The quality of the DNA was evaluated through gel electrophoresis (1.2% *w/v* agarose) (Life technologies, Madrid, Spain), and its concentration was measured using the Qubit (Thermo Fisher Scientific, Waltham, US) before storing at -20°C until 16S rRNA library preparation.
